# Avocado fruit maturation and ripening: dynamics of aliphatic acetogenins and lipidomic profiles from mesocarp, idioblasts and seed

**DOI:** 10.1186/s12870-017-1103-6

**Published:** 2017-09-29

**Authors:** Carlos Eduardo Rodríguez-López, Carmen Hernández-Brenes, Víctor Treviño, Rocío I. Díaz de la Garza

**Affiliations:** 1 Escuela de Ingeniería y Ciencias, Campus Monterrey, Tecnologico de Monterrey, Monterrey, Nuevo Leon Mexico; 2 Cátedra de Bioinformática, Escuela de Medicina, Tecnologico de Monterrey, Monterrey, Nuevo Leon Mexico

**Keywords:** Avocado fruit, Lauraceous Acetogenins, Aliphatic Acetogenins, Idioblasts, Ripening, Untargeted Lipidomics, TAG catabolism

## Abstract

**Background:**

Avocado fruit contains aliphatic acetogenins (oft-acetylated, odd-chain fatty alcohols) with promising bioactivities for both medical and food industries. However, we have scarce knowledge about their metabolism. The present work aimed to study changes in acetogenin profiles from mesocarp, lipid-containing idioblasts, and seeds from ‘Hass’ cultivar during fruit development, germination, and three harvesting years. An untargeted LC-MS based lipidomic analysis was also conducted to profile the lipidome of avocado fruit in each tissue.

**Results:**

The targeted analysis showed that acetogenin profiles and contents remained unchanged in avocado mesocarp during maturation and postharvest ripening, germination, and different harvesting years. However, a shift in the acetogenin profile distribution, accompanied with a sharp increase in concentration, was observed in seed during early maturation. Untargeted lipidomics showed that this shift was accompanied with remodeling of glycerolipids: TAGs and DAGs decreased during fruit growing in seed. Remarkably, the majority of the lipidome in mature seed was composed by acetogenins; we suggest that this tissue is able to synthesize them independently from mesocarp. On the other hand, lipid-containing idioblasts accumulated almost the entire acetogenin pool measured in the whole mesocarp, while only having 4% of the total fatty acids. The lipidome of this cell type changed the most when the fruit was ripening after harvesting, TAGs decreased while odd-chain DAGs increased. Notably, idioblast lipidome was more diverse than that from mesocarp.

**Conclusions:**

Evidence shown here suggests that idioblasts are the main site of acetogenin biosynthesis in avocado mesocarp. This work unveiled the prevalence of aliphatic acetogenins in the avocado fruit lipidome and evidenced TAGs as initial donors of the acetogenin backbones in its biosynthesis. It also sets evidence for acetogenins being included in future works aimed at characterizing the avocado seed, as they are a main component of their lipidome.

**Electronic supplementary material:**

The online version of this article (10.1186/s12870-017-1103-6) contains supplementary material, which is available to authorized users.

## Background

Avocado pulp is an excellent source of macro and micronutrients, characterized for containing highly bioactive lipids that have received increased scientific interest for their potential health-related applications. Although most attention has focused on its contents of monounsaturated oil (15–30% of total fresh weight of the mesocarp [1]), there is a recent interest devoted to a family of fatty acid derivatives called lauraceous acetogenins. Acetogenins, often present as acetylated odd-chain fatty alcohols, have shown relevant bioactivities, such as insecticidal [2–5], inhibition of acetyl-CoA carboxylase [6] and production of nitric oxide and superoxide in cells [7, 8], as well as proapoptotic effects against several cancer cell lines [2, 9–12], and recently, a promising activity against Acute Myeloid Leukemia cell lines [13], as well as sporostatic and bactericidal properties [14, 15].

Avocado fruit development can be easily divided in two different, easily distinguishable processes: fruit maturation, which is the process of growing taking place while in the tree, from 20 to 60 weeks after pollination; and postharvest ripening, comprising the softening of the mesocarp and improvement of organoleptic properties taking place only after the detachment of the fruit [16, 17]. Unlike most fruits, in which there is an initial phase of cell division followed by cell growth, in avocado fruit cell division remains active until reaching the last stage of maturation [18]. Being a climacteric fruit, during postharvest ripening an increase in respiration has been observed, coupled with ethylene production, signaling the most drastic physicochemical changes [19]. Notably, it is during fruit maturation, and not during postharvest ripening, that lipids, particularly fatty acids and triacylglycerols (TAGs) are synthesized [20], to the point that oil content is taken as a measure of fruit maturity, but not ripening stage [21].

Apart from the mesocarp, mature avocado fruit contains a single, large seed formed by one embryo surrounded by very thin seed coats; the embryo consists of two particularly large starchy cotyledons with a centrally attached very small embryonic axis [22]. In the early stages of fruit development, the embryo is surrounded by a gelatinous endosperm, which completely disappears at the final stages; thus avocado seeds are considered non-endospermic [23]. It is during these late stages that seed is separated from the vascular network of the fruit [22]. Since avocado is a member of the basal angiosperms, the oldest known flowering plants, branching away before the separation of monocots and dicots [24, 25], its seed has no defense mechanisms against dehydration [23, 26]. It has to be planted within 9 months of the separation from the mesocarp, and requires 45 days in a high humidity (>70% RH) environment for the radical protrusion to occur [26].

Another peculiarity of avocado fruit is the presence of lipid-containing idioblasts, large (~80 μm) specialized cells, distributed uniformly in the mesocarp which make up to 2 % volume of the edible portion [27] and are characterized by a thick (4 μm) wall consisting of three layers of cellulose, suberin, and lignified cellulose [28, 29]. These secretory glands are mainly occupied by a cutinized sac, topologically considered extracellular space, filled with a single, large drop of oil, and surrounded by an electron-dense cytoplasm with partially degraded organelles [27–29]. Idioblasts have been hypothesized to play a role in plant defense against herbivores [4] and fungal infections [5] mainly because their high acetogenin content [5].

Little is known about the metabolism of acetogenins in the plant, with most studies focusing on only one derivative, Persin [(Z,Z)-1-acetyloxy-2-hydroxy-12,15-heneicosadien-4-one]. Previous works have confirmed incorporation of ^14^C–labeled linoleic acid and acetate into Persin [30, 31], linking their metabolism to that of long-chain fatty acids. Furthermore, Persin concentrations were found to correlate positively with expression levels of fatty acid editing enzymes, such as ∆9-[31] and ∆12-desaturases [32], and an elongase (*avfae1*) [33]. On the other hand, a lipoxygenase (LOX) has been shown to degrade Persin in vitro, and the increase in its activity during ripening has been associated with a decrease in Persin contents [34–36]. These results suggest a connection between fatty acid and acetogenin metabolism, in both synthesis and degradation, and therefore, studying acetogenin dynamics during the most intense period of fatty acid synthesis, fruit maturation, is expected to yield useful information on this link.

Recently, we fully characterized acetogenin distribution in fruit tissues from 22 avocado cultivars from different genetic backgrounds [37]. We characterized a total of 8 different acetogenin derivatives, sampling the chemical diversity of acetogenins. Apart from generating a chemotaxonomic model, the importance of seed tissue in acetogenin metabolism was documented; and it was also hypothesized that seed and mesocarp are possibly capable of conducting independent biosynthesis of acetogenins. Furthermore, a classification system for lauraceous acetogenins was generated; three groups were proposed based on carbon numbers of their deacetoxylated backbones, which included avocatins, pahuatins and persenins (17, 19 or 21 carbons, respectively). Results from work also described in Rodríguez-López et al. favors the hypothesis that each acetogenin group may have a different fatty acid precursor. In addition, this work showed that Persin is not the main component of the acetogenin pool in avocado fruit [37]; thus, more comprehensive studies are needed considering all acetogenins in fruit to start to generating knowledge about their metabolism.

Also, although avocado high oil content has prompted several works in lipidomics characterization, most of the available literature focuses on a single developmental point, using avocado extracts as an example of a recalcitrant matrix. For example Shen et al. used non-polar extracts to test a new Solid Phase Extraction method to enrich glycerophospholipids (by retaining TAGs) and identified a total of 31 putative phospholipids [38]. Similarly, Vaikkinen et al. used avocado mesocarp to test a new ionization technique, Heat-Assisted Laser Ablation (HA-LA-ESI-MS), reaching tentative assignation of 39 features, mainly TAGs, DAGs and phospholipids [39]. Recent work by Horn et al., as a part of a proteomics experiment on avocado lipid bodies, analyzed TAG composition by MALDI-MS Imaging [40] selecting 23 features as putative TAGs, with species ranging from 48:2 to 54:1, most of which were uniformly distributed through the avocado mesocarp [40]. Noticeably, none of these MS studies coupled any chromatographic technique to the MS, and thus, did not report other lipids simultaneously, given that neutral lipids tend to mask the rest of the lipid families by force of concentration [38].

The present work was undertaken with the purpose of studying changes in profiles and concentrations of seven individual acetogenins found in the mesocarp and seed of the commercially relevant ‘Hass’ avocado cultivar, as affected by growth and postharvest ripening (Additional file 1: Figure S1). The study also aimed to characterize and contrast, for the first time, acetogenin profiles found in specialized, oil-containing idioblasts, with those of mesocarp and seed. Additionally, the effects of seed germination on acetogenin profiles and concentrations, and the effect of environmental changes on acetogenin accumulation in fruit (by comparison of samples from three different harvesting years) are described in this paper. This is also, to the best of our knowledge, the first reported use of MS-based untargeted lipidomics methodology to analyze changes of avocado lipids due to biological effectors, in this case growth and ripening, and the first work using a chromatography-coupled approach. By comparing acetogenin dynamics with the untargeted lipidomics results during different fruit growing and ripening stages, we intend to start unveiling their metabolic cues.

## Results

### Acetogenin profiles are conserved in mesocarp while seed profiles are dynamic during fruit growth

**Fig. 1:**
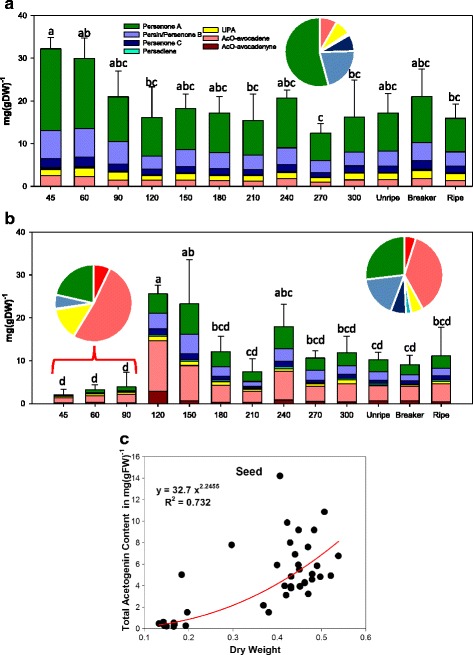
Acetogenin contents in mesocarp and seed of ‘Hass’ avocado fruit during development. (**a**) Acetogenin concentrations during maturation (left) and postharvest ripening stages (right) of avocado mesocarp and (**b**) seed tissue, grouped by fruit fresh weigth (*n* = 3) Means are the average of the corresponding biological replicates, and error bars reflect the standard deviation of the Total Acetogenin Concentrations (TAC); letters denote homogeneous group in a Tukey HSD test (α < 0.05). (**c**) Non-linear regression of TAC in seed as a function of seed dry weight during on-tree maturation

Acetogenin compounds detected in ‘Hass’ avocado mesocarp and seed are presented in Table 1, and were consistent with those previously reported for the same cultivar [37]. During growth, fruit weight increased considerably from 38 to 343 g, as did the fruit dry weight ranging from 13 to 30%. However, ‘Hass’ avocado mesocarp acetogenin contents barely changed significantly during fruit growth. There is a slight initial decay in concentrations from very small fruit to mature one (Fig. 1a); however, if acetogenin contents are expressed in fresh weight (FW) there are no significant differences among all fruit development (Additional file 1: Figure S2A). Lipid biosynthesis occurs mainly during fruit maturation rather than during ripening in avocado, and fruit weight gain highly correlated with lipid accumulation (R^2^ = 0.978, Kilaru et al. 2015); thus this apparent decrease observed when expressing concentrations in dry weight (DW) is due to the relative concentration with the increasing lipids (Additional file 1: Figure S2C &D). Remarkably, during postharvest ripening there were no differences in acetogenin accumulation (Fig. 1a, Additional file 1: Figure S2A). Acetogenin distribution was also constant in developing mesocarp (inset pie chart of Fig. 1a and Additional file 1: Table S1), always favoring Persenone A accumulation, the main acetogenin found in mesocarp (52.7 ± 4.3%), followed by Persin/Persenone B (25.8 ± 2.3%).

**Table 1:**
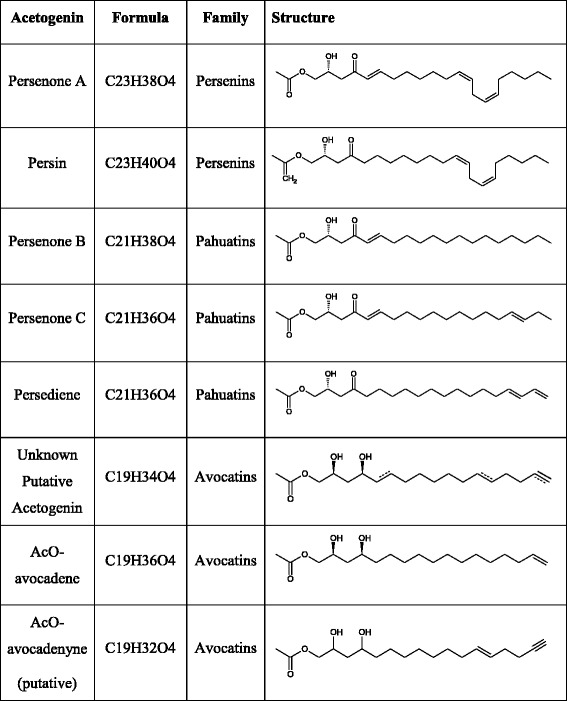
Structure of quantified acetogenins by targeted analysis

Contrastingly, seeds from avocados with fresh weights ≤90 g had very small TAC, when compared to seeds bigger than 90 g, with a 4.5-fold increase in concentrations. The same classification was suggested in the Tukey HSD grouping (Fig. 1b); considering the high variability characteristic of the tissue, with the first three stages being significantly different (group ‘d’) than the group with the highest concentration (‘a’) and the transition groups (‘b’ and ‘c’). The existence of a large group which is not statistically discernible from the low concentration group is probably due to the high biological variability characteristic of seed tissue [37] as well as the odd distribution of seed dry weight among fruits (when plotted as FW, this early group differentiated from the rest more clearly, Additional file 1: Figure S2B). On this vein, when the acetogenin concentrations in seeds were plotted against seed dry weights (Fig. 1c), a non-linear dependency can be seen ([Acetogenins] = 32.7*Dry Weight^2.246^, R^2^ = 0.732).

The distribution of acetogenin species is shown in the inset pie charts of Fig. 1b and listed in Additional file 1: Table S2. While young seeds have a low contribution of Persin/Persenone B to the pool (4.51 ± 2.96%), in mature seeds this relation more than triples (17.4 ± 3.97%); this shift is compensated by UPA, which reduces its contribution roughly two thirds (from 14.6 ± 3.31% to 5.54 ± 3.07%). In a similar fashion, avocatins (short-chain acetogenins, Table 1) contribution is greatly reduced as seed reaches maturity, with a drop on AcO-avocadenyne share from 7.85 ± 1.79% to 4.99 ± 3.49% and for AcO-avocadene from 53.7 ± 4.43% to 36.3 ± 4.88%, which was the highest percentual drop (Additional file 1: Table S2). On the other hand, the contents and profiles observed in seeds from mature green fruit remained constant during postharvest ripening (Fig. 1b).

### Idioblasts accumulate the majority of acetogenins in avocado mesocarp

**Fig. 2:**
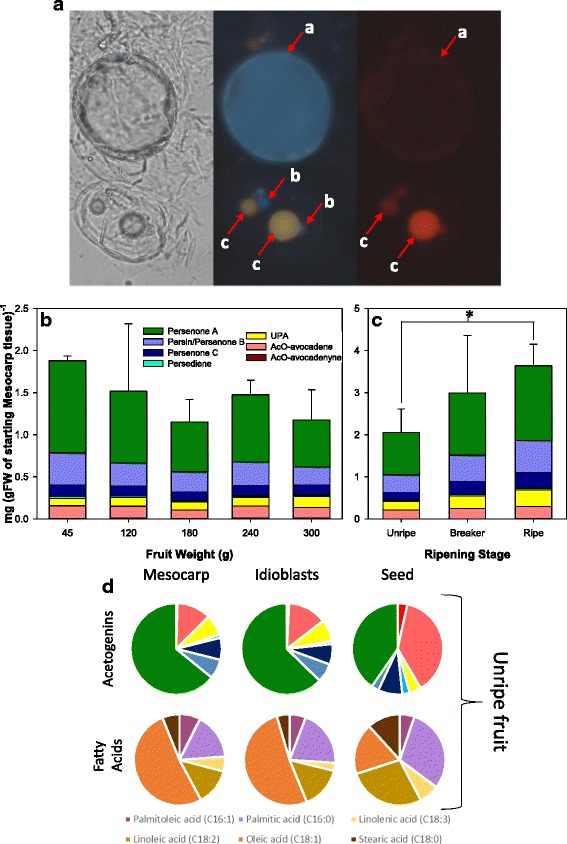
Morphological and biochemical description of Idioblasts. (**a**) Microscopy scans of digested mesocarp, dyed with DAPI and Nile Red, showing an Idioblast (top) and two parenchymatic cells (bottom) with bright field (left), and under a blue (center) and a red (right) filter. Three standing elements are marked with letters, namely the Idioblast oil sac (a), and parenchymatic cells’ nucleus (b) and oleosomes(c). (**b**) Evolution of Acetogenins concentration on Idioblasts during maturation and (**c**) postharvest ripening of the fruit; a significant difference between two postharvest ripening stages (t-test, *p* < 0.05) is shown with an asterisk (*). Bars represent the average of three measurements, and error bars reflect the standard deviation of the total acetogenins concentration. (**d**) Pie charts explaining the contribution of individual acetogenins (top) and fatty acids (bottom) to the total pool in mesocarp (left,) Idioblasts (center,) and seed (left)

Idioblast isolation was conducted on the same samples used for the fruit growth and postharvest ripening experiments. Both mesocarp and idioblast-enriched fractions from avocado fruit were seen under the light microscope, stained with DAPI and Nile Red to easily assure integrity and to observe the lipid droplet. Figure 2a, depicts idioblast cell (top) along with a parenchymatic cell (bottom); as it can be observed, apart from the difference in size (twice bigger than parenchymatic cells), idioblasts were also characterized by their highly dense cytoplasm as previously reported [28, 29]. Idioblasts were uniformly dyed with DAPI, unlike parenchymatic cells in which the dye only concentrated in a reduced location. On the other hand, the lipid-specific Nile Red staining, revealed smaller droplets in parenchymatic cells, emitting a high intensity red color, while idioblasts have a much larger droplet, occupying most of the space, glowing on a faint orange-red color. It is important to note that, while the majority of idioblasts show a single, full oil sac, some others appear to have only medium-sized droplets (Additional file 1: Figure S3). To assess the purity of idioblast-enriched fractions, Nile Red-stained idioblasts preparations were run in a flow cytometer; distribution of cells with idioblast-characteristics was at least 87.9% of the cells in the preparations (Additional file 1: Figure S4 and Table S3). Parallel to mesocarp, when acetogenins were characterized and quantified in idioblasts during fruit growth, no significant differences were found; moreover, these cells had the same acetogenin pool distribution than mesocarp (Fig. 2b and d, Additional file 1: Table S4). On the other hand, during postharvest ripening, while maintaining the same profile, idioblast TACs steadily increased (Fig. 2c, Additional file 1: Table S4), accumulating, remarkably, almost all (98.1 ± 0.8%) of the acetogenins found in ripe fruits when compared to the corresponding mesocarp replicates from Fig. 1a. Fatty acid profiles of idioblasts were also found to be the same as in mesocarp in mature green fruit (Fig. 2d). However, idioblasts accumulated only 4.1% of the saponified fatty acid contents of mesocarp (Table 2). Correspondingly, seed saponified fatty acid profiles differed from those of mesocarp and idioblasts, (Fig. 2d) with seeds having a higher share of stearic, linoleic, and palmitic acid, taking a toll on oleic acid, and had a much lower concentration of total fatty acids: 3.02 ± 0.72 mg/gFW (7.41 ± 1.96 mg/gDW). While idioblasts are reported to be present in all tissues in avocado, including seed, [29, 41, 42] the methodology used in this work did not allow us to purify idioblasts from this tissue.

**Table 2:** Fatty acid present in ‘Hass’ avocado unripe mesocarp, idioblasts and seeds

Tissue	Fatty acid (mg/gFW)						
	Linolenic	Palmitoleic	Linoleic	Palmitic	Oleic	Stearic	Total
Mesocarp	5.73 ± 0.03	8.12 ± 0.87	14.27 ± 1.56	17.77 ± 3.49	56.30 ± 8.72	6.67 ± 0.01	108.86 ± 14.40
Idioblasts^a^	0.13 ± 0.01	0.26 ± 0.08	0.69 ± 0.21	0.93 ± 0.34	2.37 ± 0.80	0.21 ± 0.02	4.59 ± 1.45
Seed	0.22 ± 0.02	0.16 ± 0.02	0.84 ± 0.13	0.90 ± 0.17	0.55 ± 0.41	0.36 ± 0.02	3.02 ± 0.72

^a^milligram per fresh weigh of the original mesocarp tissue

### Acetogenin profiles do not vary across different harvesting years nor during seed germination

**Fig. 3:**
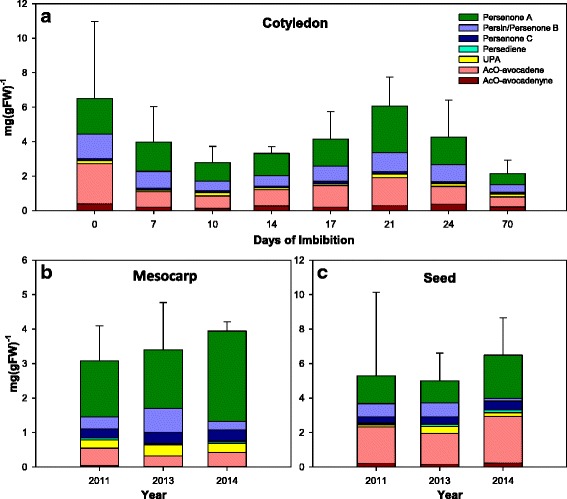
Acetogenin profiles during germination and across harvest years for ‘Hass’ avocado fruits: (**a**) Acetogenins concentration in cotyledons during germination (seedlings did not accumulate detectable amounts at any stage); and (**b**) across three years for mesocarp and (**c**) seed. Bars represent the average of three measurements, and error bars reflect the standard deviation of the total acetogenins concentration

Looking for other hints of metabolic switches for acetogenins, we monitored acetogenin levels during germination, analyzing separately cotyledons, embryonic axis (plumule and radicle) and growing seedlings using the samples collected from the harvest of 2011. Results from these experiments showed no significant trend due to the high variation in cotyledon tissue over a 70 day-period on either total acetogenins or each individual one (Fig. 3a). Surprisingly, in embryo’s plumule and radicle, or in shoots and roots from seedlings, no measurable amount of acetogenins could be detected (detection limits 0.01 mg/gFW).

**Fig. 4:**
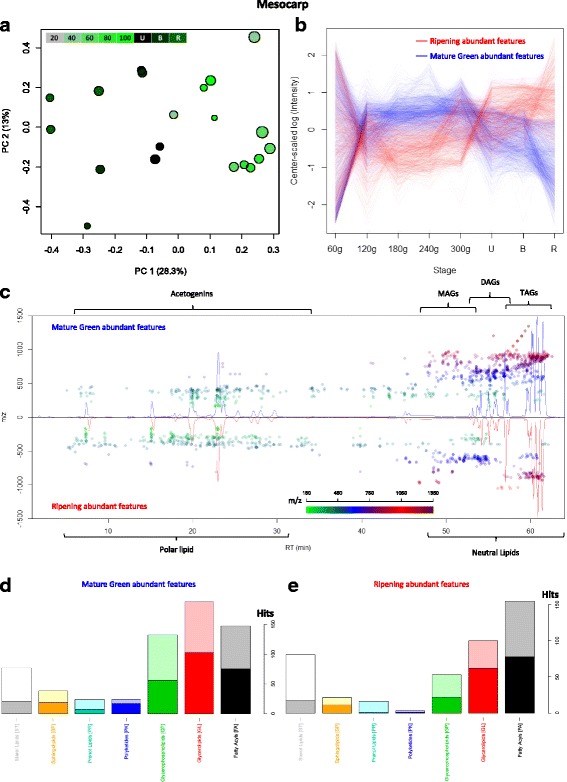
Avocado mesocarp lipidome during fruit maturation and ripening. (**a**) PCA analysis of features detected in all mesocarp samples, except the smallest stage (60 g); fruit growing and ripening stage is indicated by color and mesocarp DW is depicted by circle size. (**b**) Mean values (*n* = 3) as a function of fruit growth and ripening stage of the two divergent groups of selected features (see Additional file 1: Figure S6) from PC1, blue lines represent features that are characteristic of growing fruit and red lines of ripening fruit. (**c**) Cloud plot with chromatograms from mature green mesocarp (top) and ripe mesocarp (bottom), dots are placed above the feature elution time and their color and position represents the feature’s m/z. (**d**) Classification of features characteristic of mesocarp from growing and (**e**) ripening fruit by Lipid MAPS® Classification [45]; solid bars represent ‘fixed’ assignations and shadowed bars, ‘variable’ assignations

Additionally, to evaluate the effect of natural environmental variation in the acetogenin levels, three collections of samples at mature green stage (just harvested) over a four-year period were analyzed (Fig. 3b and c). No significant changes were observed in acetogenin accumulation in mesocarp and seed from ripe avocado fruit (Fig. 4b and c, respectively) across those years, despite differences expected due environmental stresses, given that 2013 was an atypically dry year in Uruapan, with total rain levels in the month previous to the harvest an order of magnitude less (21 mm) than the average precipitation for that month (270 mm) [43]. These changes are in accordance with a previous study conducted in avocado leaves, which revealed that while there were variations in Persin levels across 21 avocado cultivars, including several ‘Hass’ ecotypes, there were no changes resulting from leaf age or season, over a two-year period [44].

### Untargeted lipidomic analysis in avocado fruit mesocarp, idioblasts and seeds.

To gain more insight into the acetogenin and other lipids production in avocado fruit during growing and ripening, acetone extracts from selected samples of fruit growing and ripening were analyzed by HPLC-ESI-TOF. Features were detected from each tissue and analyzed by PCA; then, the most relevant principal component was chosen, and features from it were assigned possible identities by searching the most common adducts on Lipid MAPS® for preliminary identification. By doing this procedure we are presenting for the first time lipidomic profiles from each avocado fruit tissue during growth and ripening.

#### Lipidomic profiles from mesocarp show the dynamics of glycerolipids and acetogenins trough fruit ripening

Given that the difference in profiles of smallest fruits overshadows the other differences (PC1, Additional file 1: Figure S5), a PCA was performed on a subset of the samples, excluding the 60 g stage, which successfully separated samples by those that were in the ripening process from the ones that were not (Fig. 4a). Two groups were separated via score plots (top lines, Additional file 1: Figure S6) each with a particular trend, sharing a common characteristic of extremely high variability at the earliest stage, subsequently followed by either an increase in relative intensity (red, abundant in ripening) or a decrease (blue, abundant in mature green), reaching a maximum or minimum, respectively, in the ripe stage (Fig. 4b).

As it can be seen in the cloud plot (Fig. 4c), features present in mature green (top) and ripe fruit (bottom) looked similar, apart from a small section, close to the neutral lipid elution time, unrepresented in ripe fruit. Also, as mentioned above, there were more mature green abundant features (which decrease during ripening) than ripe abundant features, and the latter appeared to have lower Retention Times (RT) and m/z. When analyzing the automatic identity assignations of the features, a difference in the diversity of lipid classes was clear: lipid classes appeared to shift from Glycerolipids and Glycerophospholipids, abundant in mature green (Fig. 4d), towards Fatty Acyls, the most varied class in the ripe group (Fig. 4e). It is important to note that acetogenins are grouped by the Lipid MAPS® classification in this Fatty Acyl class [45].

**Fig. 5:**
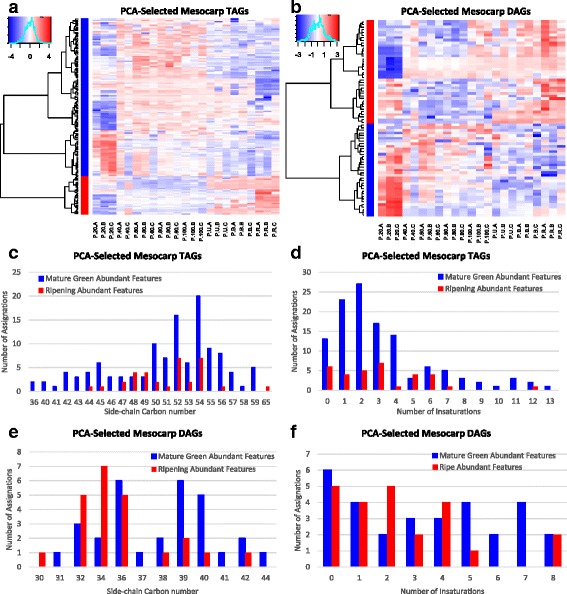
Mesocarp TAGs and DAGs dynamics through fruit develoment. Heatmaps of log-transformed, center-scaled intensities of features with manually curated identities clustered by trends, red increase, blue decrease as fruit develops. (**a**) TAGs; (**b**) DAGs. Bar plots of the assignations of Mature Green (blue) and Ripe (red) abundant features grouped by (**c**) TAG carbon number, (**d**) TAG unsaturations, (**e**) DAG carbon number, and (**f**) DAG unsaturations

Features that were assigned automatically as Glycerolipids were manually curated (Supplementary Table 5) and variations in their intensity through fruit development were plotted into heatmaps (Fig. 5a and b). It can be clearly observed that TAG diversity drops through fruit development, particularly during ripening: while 100 TAGs decreased, only 30 TAGs, increased (Fig. 5a). Interestingly, distribution of carbon number among TAGs following both trends was fairly conserved (Fig. 5c) while double bonds followed a different tendency, with monounsaturated and disaturated TAGs being the most affected by the ripening process, and saturated and trisaturated TAGs being the most common groups that increased (Fig. 5d). On the other hand, the distribution of DAGs pointed to a remodeling of the DAG pool (Fig. 5b): DAGs that decreased during ripening had a higher side-chain carbon number and unsaturations than those that increased (Fig. 5e and f). Interestingly, the number of DAGs with an even side-chain carbon number in the ripening-decreasing features was the same as those that increased during ripening (21 DAGs); however, DAGs with an odd-carbon number fatty acid are over-all decreasing, with 9 of them reaching a minimum in ripe avocado (Fig. 5b and e, and Additional file 2: Table S5).

#### Lipidome changes in idioblasts have differences in the glycerolipid remodeling to that of mesocarp

**Fig. 6:**
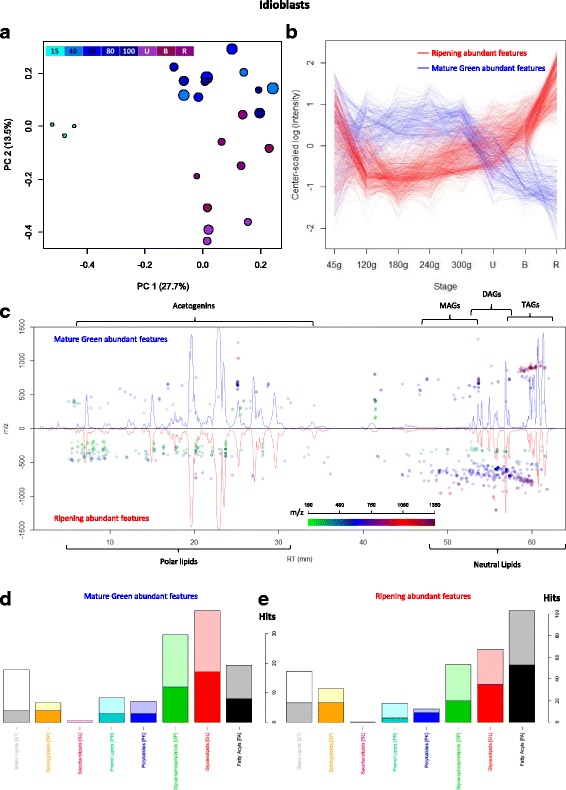
Lipidome of idioblasts isolated from avocado mesocarp during fruit maturation and ripening. (**a**) PCA analysis of features detected in all idioblasts samples, fruit growing and ripening stage is indicated by color and mesocarp DW is depicted by circle size. (**b**) Mean values (n = 3) as a function of fruit growth and ripening stage of two divergent groups of selected features (see Additional file 1: Figure S7) from PC1, blue lines represent features that are characteristic of idioblasts from growing fruit and red lines of ripening fruit. (**c**) Cloud plot with chromatograms of idioblasts from mature green mesocarp (top) and ripe mesocarp (bottom), dots are placed above the feature elution time and their color and position represents the feature’s m/z. (**d**) Classification of Features characteristic of idioblasts from growing and (**e**) ripening fruit by Lipid MAPS® Classification [45]

The idioblast-enriched fraction had a more diverse lipidome than mesocarp, with 5333 features detected. The PCA analysis showed the same separation as the PCA from avocado mesocarp: the main component separated the samples belonging to the smallest avocado triplicate, while the second component separated idioblasts extracted from avocados following ripening process from the rest (Fig. 6a). Again, only the second component was taken in consideration, and two populations were extracted from plotting the score values (Additional file 1: Figure S7). Remarkably, the features belonging to these groups followed the same pattern as those from mesocarp, (Fig. 6b). An interesting trend can be observed in the cloud plot (Fig. 6c) where mature green abundant features, which decreased during ripening, had high m/z values and were concentrated in the chromatographic region corresponding to TAGs. Ripe abundant features, on the other hand, were more disperse and had lower masses and retention times, hinting at a degradation of the aforementioned TAGs, and an increase in diversity of slightly more polar lipids. The automatic classification of the features also points to the same shift as mesocarp, with Glycerolipids and Glycerophospholipids being the most abundant classification in mature green (Fig. 6d); and, Fatty Acyls (with acetogenins in them) were the most abundant in the ripe group (Fig. 6e), confirming our observation from the targeted analysis.

**Fig. 7:**
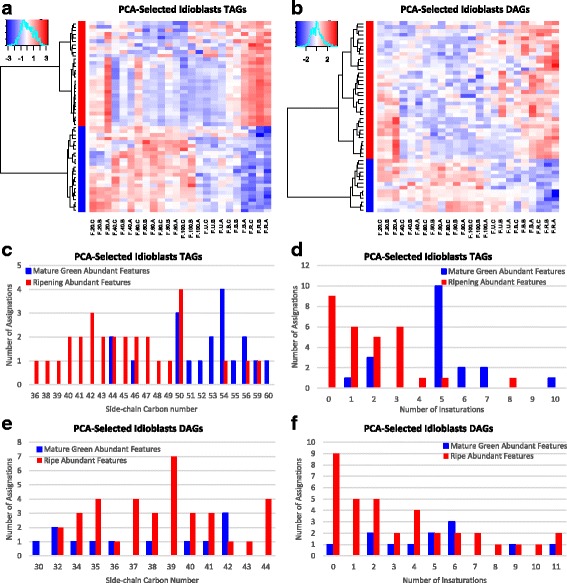
Idioblasts TAGs and DAGs dynamics through fruit develoment. Heatmaps of log-transformed, center-scaled intensities of features with manually curated identities clustered by trends, red increase, blue decrease as fruit develops. (**a**) TAGs; (**b**) DAGs. Bar plots of the assignations of Mature Green (blue) and Ripe (red) abundant features grouped by (**c**) TAG carbon number, (**d**) TAG unsaturations, (**e**) DAG carbon number, and (**f**) DAG unsaturations

When comparing lipidomic profiles, it was evident that the region of acetogenins was prevalent in idioblasts extracts while mesocarp accumulated mostly neutral lipids, confirming again the characterization made by our targeted analysis. Also, unlike mesocarp, the chemical nature of the varying glycerolipids in idioblasts differed greatly (Additional file 2: Table S6) and had encountered trends: while no trend dominated the manually curated TAGs (Fig. 7a), the majority of the DAGs decreased as fruit ripened (Fig. 7b). TAGs that decreased during ripening had higher carbon number (Fig. 7c) and unsaturations (Fig. 7d) than those that increased. DAGs, tended to have lower unsaturations as fruit ripen (Fig. 7f) and, interestingly, higher carbon number than those that decreased (Fig. 7e). Thus, they might be product of TAG degradation. Another interesting phenomenon occurs in this DAG remodeling in idioblasts, especially considering that acetogenins are odd-chain lipids: DAGs containing odd-chain FA were the most common features that increased during ripening, contrary to mesocarp (Fig. 7e). Particularly, the most abundant odd-chain category of decreasing mesocarp DAGs, it had a 39-carbon side-chain (Fig. 5e, Additional file 2: Table S5), the same as the most abundant category of increasing DAGs in idioblasts (Fig. 7e, Additional file 2: Table S6); both were also highly unsaturated (up to 8 unsaturations in mesocarp, Fig. 5f, and up to 7 in idioblast odd-chain DAGs, Fig. 7f).

#### Lipidomic profiles from seeds show glycerolipid consumption and acetogenin accumulation as main cause of changes in lipids from this tissue

**Fig. 8:**
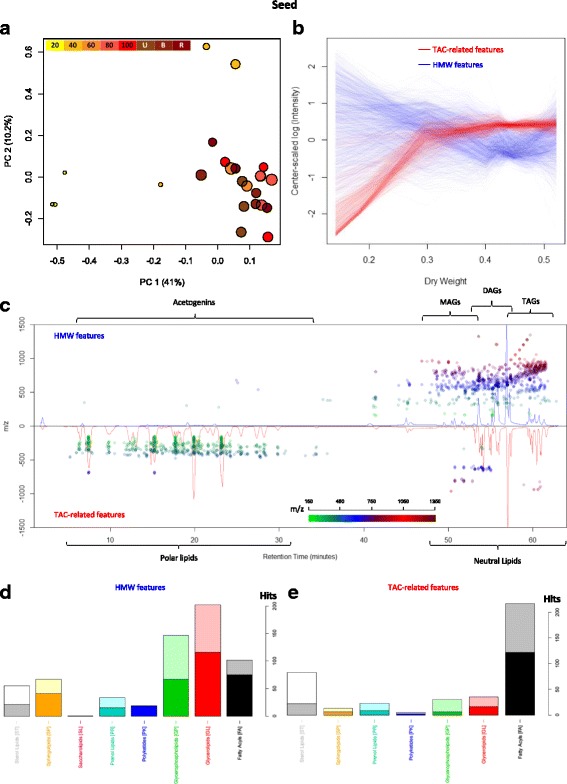
Avocado Seed Lipidome during fruit maturation. (**a**) PCA analysis of features detected in all seed samples, fruit growing and ripening stage is indicated by color and DW of the seed is depicted by circle size. (**b**) LOWESS values as a function of DW of two divergent groups of selected features (see Additional file 1: Figure S8B) from PC1. (**c**) Cloud plot with chromatograms from seeds of 15% (low DW, top) and 42% DW (high DW, bottom), dots are placed above the feature elution time and their color and position represents the feature’s m/z. (**d**) Classification of Features characteristic of small seeds and (**e**) seeds from mature fruits ripening fruit by Lipid MAPS® Classification [45]

Most of the variation found in the 3648 features detected in seed was due to features from PC1 (41%, Fig. 8a), which separated samples in an almost exact fashion as the acetogenins did (Fig. 1c). In fact, the acetogenin contents per dry weight (TAC-DW) could be effectively predicted just by the value of the main component in the sample (R^2^ = 0.91, Additional file 1: Figure S8A). This suggests that the most relevant changes occurring in the seed lipidome during fruit growth are due to accumulation of acetogenins, or acetogenin-related metabolites, as seed dry weight increases.

The two main groups responsible of the separation by TAC-DW differed greatly in mass, with most of the scores relating positively to TAC-DW having low m/z, and features with high-intermediate m/z being on the negative side of the scores plot (Additional file 1: Figure S8B). TAC-related features (Fig. 8b, red lines) presented a clear common trend when plotted by stage (Additional file 1: Figure S8C). This trend showed an analog behavior to that of the barplots in Fig. 1b, increasing rapidly in the first stages and reaching a plateau at medium-sized avocadoes; similarly when plotted against DW, with the plateau being reached at about 30% dry weight (Fig. 8b). On the other hand, the trend of the features with high molecular weight (Fig. 8b, blue lines) follows a decline as fruit grows, which, when plotted against dry weight, reaches a minimum at 45% dry weight (Fig. 8b). The cloud plot in Fig. 8c shows the typical lipidome profile from small seeds (bottom) and mature seeds (top). Features in the region of neutral lipids (high molecular weight, longer RT) were more abundant in seeds from smaller avocados, and features in the acetogenin region (low m/z and short RT) were more abundant in mature seeds (Fig. 8c). On the other hand, there were few metabolites in the region of polar lipids of high molecular weight that were more abundant in mature seeds. When analyzing Lipid MAPS® assignations, these assumptions were further supported, given that the most common putative identity of the features belonging to the high molecular weight group (Fig. 8d) was the Glycerolipids class. In contrast, the most common assignation, by far, of the TAC-related features (Fig. 8e) was as Fatty Acyls.

**Fig. 9:**
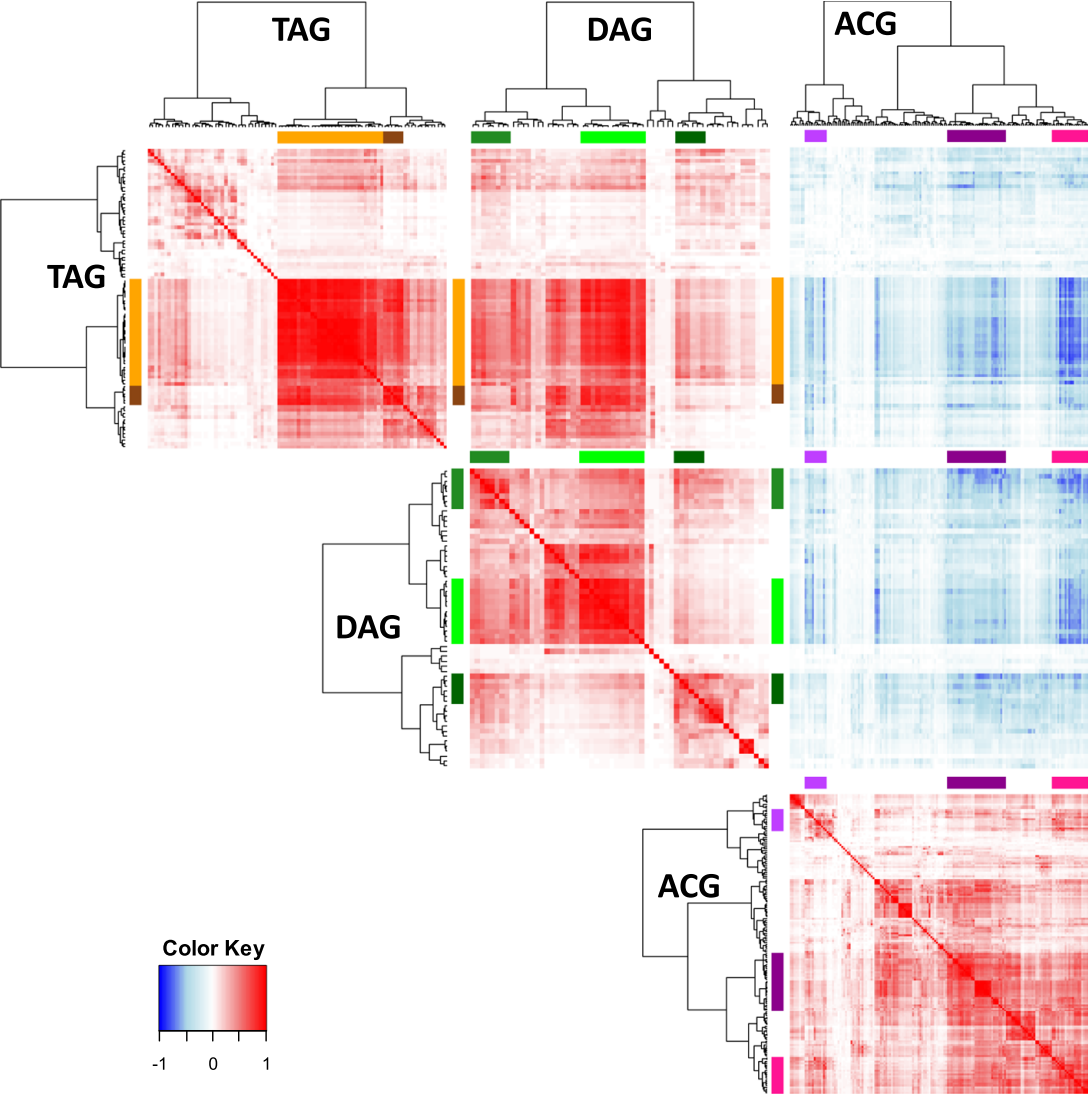
Correlations among TAGs, DAGs, and acetogenins from seed tissue. Heatmaps show Pearson correlation coefficients times to the 5th^,^; dendrograms show the lustering of the features of the same class against themselves. Positive correlations are depicted in red and negative correlations in blue

A further manual curation of the assignations was performed, to better characterize the metabolites that had the most relevant changes (Additional file 2: Table S7). Heatmaps of these curated assignations evidenced the decline in TAGs and DAGs as the seed develops, this in complete contrast with the increase in acetogenins (Additional file 1: Figure S9). In total, 139 features assigned as Glycerolipid decreased as seed matures (Additional file 2: Table S7); the most abundant group was TAGs, with 71 putative identity assignations; then DAGs, with 51; MAGs with 3; and 14 assigned as other glycerolipids. Most of these glycerolipids were polyunsaturated, and interestingly, more than one third of the putative TAGs and DAGs had either one or three odd carbon-number fatty acid radicals (Additional file 2: Table S7), although since this method cannot differentiate glycerolipids with a pair of odd carbon-number fatty acids, this number may be greater. A targeted analysis of adducts and fragments of the TAC-related features revealed a group of features sharing signature characteristics with the acetogenin NMR-confirmed standards, such as high oxygen count (O ≥ 4), odd number of carbon atoms, and loss of the acetoxy group, along with the fact that their scores on the PC1 were high enough to be selected (Additional file 1: Table S4). A total of 163 features were assembled in 35 groups that were assigned putative identities as acetogenins, 30 of which were new to us (Additional file 2: Table S7). Some of these putative acetogenins would have characteristics that have not been previously reported, such as completely saturated Persenins, triple acetoxylations, and highly polar acetogenins with short retention times (Additional file 1: Table S4). Therefore, it is reasonable to claim that as seed increases in dry weight, glycerolipid contents, particularly TAGs and DAGs, decrease, while acetogenins and acetogenin-related metabolites increase. This observation is further supported with correlation analyses (Fig. 9), in which it is very clear the very high negative correlation of acetogenins with both TAGs and DAGs. Interestingly, when analyzing the highest correlations between TAGs, DAGs, and acetogenins, there is a cluster of the former group that has the highest correlation with acetogenins, rather than DAGs.

## Discussion

Little is known about aliphatic acetogenin metabolism in avocado. Apart from its response to pathogens [34], elicitation with ethylene and discovery of linoleic acid as one precursor of Persin [30, 31]; there have been no further studies about the production and distribution of these metabolites in avocado fruit. As a first approach we fully characterized acetogenins produced in 21 different avocado accessions [37], showing that this is a very conserved metabolism in *Persea americana*. The current study was undertaken to further characterize acetogenins in the most produced cultivar worldwide, ‘Hass’. We followed acetogenin and lipid production in mesocarp, idioblasts and seeds from avocado fruit during maturation, ripening, seed germination and harvesting years looking for indication of possible metabolic switches that can serve as a system to further characterize acetogenin biosynthesis. In doing this we have produced for the first time full acetogenin profiles during fruit growth and, moreover, to the best of our knowledge we have generated the first LC-MS based lipidomic profiles of tissues of this fruit mostly known for its oil accumulation.

### Mesocarp and idioblasts lipid metabolism differs greatly during avocado fruit ripening

The growth of avocado fruit was not a clear differential factor for acetogenin contents in mesocarp and idioblasts. Early works followed only two acetogenins during fruit maturation from the cultivar ‘Fuerte’, Persin [5, 34] and AcO-avocadene [5], which represent respectively 27 and 12% of the total acetogenin pool in mature green ‘Hass’ cultivar fruits [37]. AcO-avocadene did not have a significant change in ‘Fuerte’ mesocarp as the fruit expanded (up to 5-fold increase in fresh weight) [5], which is in accordance to our findings (Fig. 1a). On the other hand, it was reported that Persin contents decreased as fruit expanded [5] and during ripening [36]. The co-elution of Persin/Persenone B did not significantly change during development in avocado mesocarp. However, since Persenone B has an absorption maxima closer to 220 nm than Persin [37] it is possible that it is masking changes in Persin concentration during development.

The main features that changed in mesocarp as fruit ripens were TAGs and DAGs. Interestingly, the level of polyunsaturation decreased in TAGs during ripening, with DAGs having more hits with two and four desaturations following the opposite trend, increasing during ripening. There is only one report about changes in avocado lipids during ripening: the mesocarp of ‘Hass’ avocados showed that the unsaturated linolenic, linoleic and palmitoleic acids slightly increased during eight days after harvesting, while the main avocado FA, oleic acid, remained constant [21]. It its possible then that the increase in polyunsaturated DAGs is related to this observed trend, probably as intermediates in the catabolism of TAG reserves and not de novo synthesis, as it has been previously suggested that lipid accumulation stops as fruit is harvested [46, 47]. On the other hand, and very interestingly, in idioblasts cells there appears to be an increase in chemical diversity as fruit ripens, as the features that increase during ripening more than double those that decrease. Remarkably, we found that almost all total acetogenins in the ripe fruit were stored in these specialized cells, which only account for 2% of the mesocarp volume [27], this suggests that idioblasts are the main site of acetogenin synthesis within mesocarp.

It has been shown that isolated idioblasts are able to synthesize Persin when elicited with ethylene [30] and previously it was roughly estimated that 70–90% of the Persin was accumulated in idioblast from mature avocado mesocarp [5]. In addition, formation and maturation of lipid-containing idioblasts seem to be independent from the fruit developmental stage (idioblasts from all stages in their development have been detected from small ovularies to unripe immature, unripe mature and ripe fruits) [28, 29, 41]. Therefore, the measurement of whole mesocarp fatty acid contents would mask the contribution of the small amount of lipids present within idioblasts for acetogenin biosynthesis; this is why we did not find a correlation between oil accumulation and acetogenins during fruit growing and only when normalizing by dry weight did acetogenins seem to slightly decrease in mesocarp (Fig. 1a). Nevertheless, it could be argued that idioblasts can be a place for acetogenin storage rather than the place of biosynthesis, we consider this possibility as low for the following reasons. In young fruits, protoplasts of idioblasts react immediately with colored stains, while in mature fruits idioblasts generally remain impervious, probably because of suberin deposition [29, 41]. Also, idioblasts do not lose integrity during ripening because the suberin layer is not degraded by neighboring enzymes [41]. It is probable thus, that in immature fruit idioblasts can interchange molecules with parenchymatic cells. However, we have found more acetogenins in idioblasts from ripe fruit when they are closed and would not be able to translocate acetogenins (Figs. 2 and 3). We cannot rule out that parenchyma cells could be another -minor- place of acetogenin biosynthesis, but evidence shown here indicates the majority of acetogenin biosynthesis happens in these specialized cells in avocado mesocarp.

### Acetogenins are likely produced independently in avocado seeds and their accumulation is triggered during early fruit growth

Untargeted lipidomic analysis of seed tissue set the importance of acetogenins in the developing seed, revealing them as one of the main lipid classes by placing this oft-ignored compounds in the context of the rest of the lipidome. Lipids have been characterized in mature ‘Hass’ avocado seeds previously [48], and the total lipid extraction reported to be 11 ± 1.5 mg(g FW)^−1^, with a composition mainly consisting of neutral lipids (80.3%) followed by glycolipids (12.3%) and phospholipids (7.4%); the latter distribution calculated as percentage of the total eluate from a silicic acid column. The same study also declares TAGs as the main component (around 75%) of the neutral lipids, followed by DAGs (~ 10%) and MAGs (~5%) [48]; which resembles the distribution of the features found here to be relevant in seed development. However, here we report acetogenins as one of the main components of ‘Hass’ avocado seed lipids, with an average composition in mature green fruits (4.99 ± 1.61 mg[g FW]^−1^) in the same order of magnitude as the total saponifiable fatty acids (3.02 ± 0.72 mg[g FW]^−1^), which would make a significant fraction of the total lipid extract reported. A visual analysis of the chromatograms presented here of seed lipid extracts revealed acetogenins as one of the main contributors to the seed lipidome, particularly in seeds coming from mature green avocados (Fig. 8c, bottom chromatogram). This was further supported by the unsupervised analysis, that revealed that acetogenins (tagged as fatty acyls) were responsible for the main variations in seed during dry weight accumulation, as they increased as seeds reached maturity, while glycerolipids, the main peaks in immature seeds, decreased. In fact, very small seeds presented a visually distinct lipidomic profile than those from fully-grown fruits, coinciding with their very distinct anatomy: here we are profiling lipids from seed coats, endosperm and small embryo while in mature seeds we are profiling lipids mostly from embryo’s cotyledons, the tissue that grows the most during avocado seed development, mature seeds consist more than 99% cotyledon tissue. Other studies could not assess this as seeds lipids have been characterized only by targeted analyses and just in one sample of purchased mature fruit [38, 48, 49].

We found that the main groups of seed lipids that changed through development corresponded to Glycerolipids (neutral lipids + glycolipids) and Glycerophospholipids, which decreased as seed embryo increased in dry weight, and fatty acyls, mainly acetogenins, which were by far the main lipid class enclosed in features that reached a maxima in mature seeds. The latter confirms the observation of the targeted approach, and indicates that the acetogenin metabolism is quite dynamic in this tissue, and probably influenced by substrate, given they present a dry weight dependent accumulation. Moreover, avocatins (C19) are highly concentrated in seed when compared with mesocarp and idioblasts (Fig. 1b, inset, [37]), which coincides with the fatty acid distribution with a higher contribution of palmitoleic acid (C16) in seed than in mesocarp and idioblasts. Interestingly, more than one third of the Glycerolipids that decrease through development have side-chains consisting of either one or three odd-chain fatty acids. It has been reported that, in ripe fruits, odd-chain fatty acids are present in avocado seed oil extracts in a higher percentage than in mesocarp, although in trace quantities [50]. It is therefore expected that in immature seeds, these fatty acids will be present in biologically relevant quantities. Also, since they decreased as acetogenins increased in concentration, the former are the main candidates to being precursors of the latter.

In most avocado cultivars, mature seeds generally accumulate more acetogenins than mesocarp [37]. We have previously hypothesized that this tissue might be capable of an independent synthesis, since it has distinct profile of both acetogenins [37] and fatty acids [50] than mesocarp. It was then very interesting to find here that seeds from very young fruit accumulated acetogenins in minute amounts and with different profiles than seeds from more developed fruits. As previously mentioned, seeds from the smaller fruit samples presented the characteristics of early-immature avocado seeds, which included a predominant gelatinous endosperm tissue covering the embryo and two thick and fleshy seed coats [22]. Conversely, in more mature seeds, the embryo grows steadily while the endosperm tissue disappears and the seed coats shrivel and darken until dry; thus, hindering the translocation of molecules between embryo and pericarp [22]. It was then (at weights >90 g) when acetogenins began to accumulate significantly in our samples; thus, acetogenin accumulation in seeds correlated negatively with endosperm abundance and vascular continuity and positively with the embryo growth. Then, it is tempting to speculate that acetogenin accumulation in seeds may be either triggered to relocation of resources during seed development, or its accumulation would be exclusive to embryo, particularly cotyledons (embryonic axis showed no detectable acetogenin accumulation), which displace the endosperm in later stages of maturity. Correspondingly, because acetogenins accumulated the most when the transfer of molecules seemed to be obstructed by the drying of seed coat vascular system, it is highly probable that cotyledons are capable of independent biosynthesis. Furthermore, it has been recently reported that genes involved in linoleic acid biosynthesis show higher expression levels in mature avocado seed tissue than in fruit [47]; however, mature seeds accumulate minute amounts of this (and every other) fatty acid. Particularly, Fatty acyl ACP thioesterase (FatB) important for long-chain fatty acids export from the plastid, Ketoacyl-CoA synthase (KCS1), in charge of activating and making them available for enzymatic reactions, and Oleate Desaturase (FAD2), which forms C18:2, were expressed more than 4, 3 and 2-fold, respectively, in seed than in avocado mesocarp [47]. Given that linoleic and linolenic acids, are orders of magnitude less accumulated in mature seed than in pulp (17 and 26-fold less, Table 2), we speculate that such overexpression of long-chain fatty acid biosynthesis genes is directly related to acetogenin production, as we have shown the relevance of these metabolites in seed tissue. Evidence presented in this work, along with this previously reported information, strongly supports our hypothesis of seeds -particularly cotyledons- being able to independently synthesize these metabolites.

The trigger of acetogenin accumulation in seeds is the clearest indication of a possible switch that we have found in our studies, as acetogenin profiles are quite constant among avocado cultivars [37], changed little during growth and ripening in mesocarp, and environment seemed not to significantly affect them either. On the other hand, during germination, acetogenins from cotyledons did not change while embryonic axis and seedlings were not able to produce detectable amounts of these metabolites. As acetogenins are present in avocado leaves [44], it is highly probable that a similar switch exists also in this tissue, probably coinciding with the shift from auxo- to autotrophy. Therefore, this tissue offers an attractive model to study acetogenin biosynthesis as it seems that at one point during maturation, most of the seed lipid reserves are used to produce them.

### Acetogenin building blocks are likely to come from TAG editing and catabolism in avocado idioblasts and seeds

It has been shown that linoleic acid is incorporated to Persin [30, 31] and that the expression of some FA editing enzymes positively correlates with this single acetogenin [31, 32]. This evidences FAs as precursors, however, until now we can point the metabolic process in which acetogenin biosynthesis might begin. Here we showed that acetogenin contents increased during fruit ripening in idioblasts; in the same manner, the most common DAGs that also increased during ripening, had odd-carbon, highly unsaturated fatty acid chains, while long-chain highly processed TAGs decreased. Seed lipidome showed the same trends ever more clearly, as it seemed that TAGs and DAGs pools were committed to acetogenin synthesis during seed development.

TAG biosynthesis and FA β-oxidation processes have been fully described in plants; however, the steps in TAG catabolism remain elusive. Forward and reverse genetic studies in Arabidopsis have shown that TAG catabolism is triggered by SUGAR-DEPENDENT1 (SDP1) and SDP1-LIKE lipases [51, 52]. In fact, sdp1 transcript abundance increases at the beginning of seed maturation and it is expressed the most in late development; this coincides with the decline in TAG that we observe here in avocado seeds. Other lipases that must act on DAGs and MAGs to produce free FA and glycerol are yet to be characterized. The free FAs need to be activated to an acyl-CoA and translocated to the peroxisome for β-oxidation [53]. Evidence produced by this work suggests that the biosynthesis of acetogenins happens precisely in between these two processes. Moreover, very interestingly, it has been reported that besides SDP1 lipase, there is a lipoxygenase-dependent degradation of storage lipids in some plants [54]. LOXs (linoleate:oxygen oxidoreductase), in specific 13-LOX, can prime the hydrolysis of polyunsaturated TAGs by oxygenating their esterified FA residues [54, 55]. These LOXs produce a wide array of oxygen-rich lipids, to the point where acetogenins may fall within the chemical diversity of their products. Some of the LOX-dependent reactions which may be taking place are side-chain hydroxylations, as well as production of α and γ-ketols [56], also, degradation of LOX-derived oxylipins leads to β-ketols [57] similar to the polar head of acetogenins (Table 1), and LOXs produce keto-PUFAs [54], which have a *cis* double bond adjacent to the keto group, the same as Persin and other acetogenins. In this paper we report several putative acetogenins containing high number of oxygens (Additional file 2: Table S7), which may correspond to intermediary products with side-chain hydroxylations. Unfortunately, our current approach is database-dependent; therefore, unreported molecules are virtually impossible to identify. Even though, we dare to speculate that acetogenins, −oxygen-rich aliphatic molecules- could start to be produced by editing enzymes acting directly on TAGs and maybe DAGs, rather than on free fatty acids or other fatty acyl-ester moieties in avocado.

## Conclusions

For the first time five different acetogenins, plus two tentative aliphatic acetogenins were simultaneously analyzed in avocado mesocarp, seeds, and specialized idioblast cells, during fruit growth, postharvest ripening, germination, and three harvesting years. This is also the first record of a chromatography-coupled, untargeted lipidomics approach at analyzing avocado fruit.

By contextualizing acetogenins in the seed lipidome, our results reveal their importance as a major lipid family in seeds, not only by being one of the most accumulated lipids by weight in mature seeds, but also responsible for the main variations in lipid metabolites as seed matures. This dry weight dependent increase in acetogenins coupled with a decrease in TAGs and DAGs (a third of which have odd-chain fatty acids), along with previously reported high expression of fatty acid synthesis genes in this tissue, supports our claim that seed is independently able to synthetize acetogenins in cotyledons and places their synthesis during oil catabolism. The 35 putative acetogenins found in seeds by the lipidomic analyses shows the high diversity of these compounds and evidences the ramifications of this metabolism in avocado. In addition, the prevalence of acetogenins in the avocado lipidome hints to an important yet to be discovered role of these metabolites in the physiology of the plant.

Lipidomic profiles from mesocarp and idioblasts showed the complexity of the idioblast lipidome, and corroborated the observations from our targeted analysis. In addition, it unveiled the dynamics of lipids during fruit growing and ripening showing that mesocarp and idioblasts, although following the same general tendency, differed on trends within specific subfamilies, such as TAGs and DAGs. Idioblasts were shown to accumulate most of the acetogenins in mesocarp, while only accounting for a minimal fraction of the total fatty acids. They thus become the main candidate for being the place of acetogenin biosynthesis in mesocarp. Characterization of this cell-type will definitely help to describe acetogenin metabolism in avocado.

All the evidence presented here will serve to continue to study acetogenin metabolism focusing on idioblast and seeds and also shows for the first time lipidomic profiles from avocado tissues that evidence the prevalence of these compounds in avocado. Also, this work sets strong evidence for acetogenins being included in all future work aimed at characterizing the avocado seed lipidome, as they are a paramount component of the lipid fraction.

## Methods

### Plant material

Avocado fruits from the ‘Hass’ cultivar were harvested from orchards located in Uruapan, Michoacán, México (19°25 N, 102°03 W; 1620 m AMSL), which is the major commercial avocado producing region in the country. Samples for the different experiments were collected and studied between the years of 2011 to 2014. After collection, avocados were kept at ambient conditions in perforated bags overnight and shipped in closed containers with activated carbon. Samples for the first studies, described below, were collected in 2013 and upon arrival to the Centro de Biotecnología FEMSA, avocados were divided in three subsets for the fruit growth (Study I), postharvest ripening (Study II), and idioblast isolation studies (Study III). As it is described in the following sections, other avocado samples were also analyzed in Studies IV and V to study the effects of seed germination and harvesting season on acetogenin profiles, respectively.

*Study I. Fruit growth* – To characterize changes during fruit growth (Additional file 1: Figure S1, first part) avocado fruits were selected and separated directly at the Uruapan orchard by weight. Samples were grouped by two methods: by fruit fresh weight, to assess changes related to growth, into 10 categories (45, 60, 90, 120, 150, 180, 210, 240, 270 and 300 g) containing three replicates each; and by dry matter, to investigate relation to oil content. Upon arrival at the laboratory, the selected avocados were stored at 4 °C for 48 h, followed by freezing at −20 °C for other 2 days, and final storage at −80 °C.

*Study II Postharvest ripening* - Fully developed avocados (300 g), harvested at the same time than those for Study I were placed under a temperature controlled, ventilated environment until reaching one of the three sought ripening stages, in order to assess postharvest evolution of lipids (Additional file 1: Figure S1, second part). Fruits were separated by hedonic scale in Unripe (one week after detachment; peel still green); Breaker (two weeks after detachment; peel half black, half green, mesocarp still hard but softening) and Ripe (two and a half weeks after detachment; peel black and mesocarp soft, ready to eat); each stage with three replicates. When samples reached each stage, freezing and storage was followed as described in Study I.

*Study III. Idioblast characterization-* Idioblast isolation was also conducted on the same samples used for Study I at every other stage during growth, and from Study II at all postharvest ripening stages, to characterize acetogenin contents and distribution; and finally from 5 mature green (300 g) avocados to simultaneously assess acetogenin and fatty acid profiles. To avoid compromising cell integrity, idioblast extraction was performed as soon as avocados were at the desired stage. Samples were processed into slices, digested and fractionated to isolate idioblasts as described below; and the remaining tissue was frozen at −20 °C until further analysis. Thus, each idioblast replicate has a corresponding mesocarp and seed counterpart from Studies I and II.

*Study IV. Germination studies*- A set of samples collected on October 2011, were used in seed germination studies. Germination was conducted as reported [26] with minor adjustments. Briefly, avocado seeds from fully ripe black fruit were washed with soap and stored for three days at 4 °C in closed plastic bags filled with sterilized peat moss, to avoid dehydration. Prior to germination experiments, seeds were rinsed, decorticated (to remove seed coats) and a horizontal cut was made at the base of the embryo, without damaging the embryonic axis. Cut seeds were placed in a container filled with water covering one quarter of the seed. Samples were changed to individual Magenta™ vessels as they grew, and germinated inside a growth chamber (23 °C, 18:6 light/dark cycle). Embryonic axis and cotyledon sub-samples were collected in triplicate at the beginning of the experiment (day 0), twice a week after germination during a 3-week period (days 7 to 24), and after 10 weeks of imbibition (day 70). Embryonic axis (plumule and radicle) and cotyledon sub-samples were flash-frozen in liquid nitrogen, and stored at −80 °C until further analysis.

*Study V. Seasonal effects-* To investigate possible changes in acetogenin profiles throughout the harvesting years, sampling was conducted on October 2011, June 2013 and April 2014. Avocado fruits used for the study were collected at a mature green stage and stored as described above. Studies I, II, and V included acetogenin determination for both fruit mesocarp and seed tissues.

### Determination of moisture content

Dry weight was determined by weighting 5 g of material (seed or mesocarp), cutting it in thin slices (mesocarp) or small cubes (seed) and incubating at 105 °C until constant weight was achieved (typically 5–6 h) [58].

### Idioblast fractionation

Idioblasts were fractionated as described elsewhere [29, 59], with modifications. Briefly, 5 g of avocado mesocarp, in a slice, were cut to small pieces and briefly homogenized at 11000 rpm in 10 mL of a buffer containing 10 mM MES, 100 mM sorbitol, 1 mM CaCl_2_, 0.2% BSA and 0.2% DTT, at pH 5.5, and lytic enzymes (Cellulase Onozuka RS, 165 units/mL; and Macerozyme R10 mix, 15 units/mL, final concentration; Phytotechnology Laboratories). Oxygen was removed from the headspace with Nitrogen and the homogenate was incubated for 2 h in the dark at 150 rpm and room temperature. Afterwards, the mix was briefly homogenized again, and then filtered through nylon mesh filters of pore size 140 and 61 μm, and washed in the filter with buffer without enzymes. Fraction F140 contained mainly vascular tissue and undigested tissue, and fraction F60 was the idioblast-enriched fraction. Cell integrity and purity (absence of parenchymatic cells) were checked by microscopy, which clearly differentiates intact from burst idioblast cells and also lipid-containing idioblast from parenchymatic cells (described below).

### Acetogenin extraction

Extractions were made as described previously [37], namely, tissues were separated, mesocarp (2 g) was cut in even, longitudinal slices, and cotyledons (1 g) were macerated while frozen. On the other hand, idioblast enriched fractions (recovered from 5 g of mesocarp) were analyzed directly. Extraction was achieved by addition of 15 mL of acetone, where samples were homogenized with the aid of a Polytron homogenizer (Ultra-Turrax T25, IKA-Werke, Germany) for 3 min, sonicated for 1 min and clarified by centrifugation at 10000 g at 25 °C for 10 min. A 1 mL aliquot was then taken and dried under nitrogen, redissolved in 2 mL of water and added 2 mL of dichloromethane, and the organic phase was recovered, dried, resuspended in 1 mL isopropanol and filtered through a 0.2 μm PTFE filter, for HPLC injection. The extraction was made under dim light and for every step following homogenization, air was displaced from the headspace using nitrogen gas.

Extracts were separated with the aid of a C18 column (Zorbax Extend-C18, 3x100mm, 3.5 μm; Agilent, CA, USA) using a HPLC-VWD (Series 1100; HP, CA, USA) system and a gradient elution program using water (A) and methanol (B) as mobile phases, as stated in [37] only with a minor modification to column temperature, set to 35 °C. Chromatographic profiles were obtained by measuring absorbance at 220 nm and identities were assigned by comparing the retention times to those with NMR-confirmed, purified peaks by Rodríguez-Sánchez et al. [60]. Calibration curves were generated for every purified compound based on weight, except for Persenone A, for which an extinction coefficient is available. Only peak **(3)**, an Unknown Putative Acetogenin (UPA), was quantified in Persenone A equivalents. Since Persin co-eluted with Persenone B [37] the chromatographic peak was considered as both Persin and Persenone B, and quantified with a Persenone B calibration curve, as it is the moiety that absorbs the most at 220 nm (Persin absorption maximum is at 208 nm [37]).

### Fatty acid extraction

For lipid extraction, a modified Folch method was used [61] in which the tissue or an idioblast fraction (0.5 g) was homogenized in a 2:1 solution of dichloromethane:methanol (10 mL) for 3 min, sonicated for 5 min, and left at room temperature for at least 10 min before centrifugation (10,000 g) at room temperature for 5 min. Clarified phase was then vigorously mixed with a NaCl solution (0.9%, 2 mL), then centrifuged (5000 g, 2 min) and the recovered organic phase was evaporated. The remaining oil was then resuspended in a KOH solution (4 mL, 1 M in 96% ethanol) and left overnight at room temperature, under a nitrogen atmosphere, for saponification. The solution was then mixed with water (10 mL), and extracted 3 times with hexane-diethyl ether (1:1, 10 mL). Organic extract was further washed with water (10 mL), which was then mixed with the previous aqueous phase and acidified with HCl to a pH of 3. Fatty acids are recovered from acidified phase with subsequent extractions (10 mL, 3 times) with hexane-diethyl ether (1:1). Organic extracts were evaporated to dryness, re-suspended in isopropanol and passed through a PTFE filter (0.2 μm) prior to injection.

Separation and detection were made by HPLC-ELSD (1200 Series; Agilent) with the aid of a Luna C8(2) column (2.6x75mm, 3.5 μm; Phenomenex) using the vendor application No. 1258 [62] with slight modifications. Solvent gradient was programed to change from 70% acetonitrile in water, to 90% acetonitrile during the first 10 min, followed by a change to 100% acetonitrile by minute 11, and kept for 4 extra minutes, before returning (at minute 15) to the initial conditions for 5 min before the next injection, all at a flow rate of 0.3 mL/min. Detector was set to a temperature of 40 °C, with a gain of 4, with no offset, and a sampling rate of 0.1 s with a gas pressure of 3.3 bars; quantification was made by comparing areas to a curve made with certified standards for each fatty acid (Palmitic, Palmitoleic, Stearic, Oleic, Linoleic, and Linolenic acids), which were purchased from Sigma-Aldrich (St. Louis, MO, United States).

### Staining experiments

For staining experiments, nucleic acids were stained using 4′,6-diamidino-2-phenylindole (DAPI, ThermoFisher, USA), and a lipid specific dye, Nile Red (Sigma-Aldrich, USA), was used for oil staining following vendor instructions. Idioblast cell integrity was visualized in an AXIO Imager.A2 Microscope (Carl Zeiss, Oberkochen, Germany) with a HXP 120C UV source (OSRAM, Munich, Germany) equipped with a mercury lamp. Cytometric measurements on stained samples of pulp homogenate, idioblast enriched and permeated fractions were performed on a BD FACSCanto II flow cytometer (BD, San Jose, Calif., U.S.A.). Data was acquired from a total of 10,000 events per sample, collected at low flow rate through channels PerCP (670 LP nm band-pass filter) and FITC (530/30 nm band-pass filter), in forward and side scatter. Group discrimination and purity assessment was performed in R, as stated in the Data Analysis section.

### Lipidomic analysis

All acetone extracts from *Study III* (Idioblast characterization) were selected to follow the lipidomics pipeline, along with their corresponding extracts from mesocarp and seed in *Study I* and *II*, with the exception of the smallest stage (45 g) for which there was not enough sample and was substituted by the next stage (60 g) in mesocarp and seed. Acetone was evaporated in the dark, under vacuum, at 45 °C, until dryness, and resuspended in Isopropanol. Resuspension volume was calculated as to inject a constant amount of dry weight for each tissue.

#### Chromatographic separation

Extracts were separated with a Luna C18(2) column (150x2mm, 3 μm; Phenomenex, CA, USA) using a HPLC (Series 1100; Agilent, CA, USA) coupled via ESI to a TOF MS Detector (G1969A; Agilent, CA, USA) system and a gradient elution program that included a water:Acetonitrile mix (4:1 *v*/v; phase A) and an Isopropanol:Acetonitrile mix (9:1 v/v; phase B) as mobile phases, both modified with 10 mM Ammonium Acetate and 0.1% Formic Acid. Samples were separated at 55 °C and the elution gradient had a constant flow of 0.2 mL/min. The 65-min gradient consisted of linear ramps from 40% to 43% B (6 min); jumping to 50% B at minute 6, and ramping linearly to 54% B until minute 36; then changing immediately to 70% B and linearly increasing to reach 99% B by minute 54. This condition was kept until minute 55, when column returned initial conditions (40% B) where it equilibrated (10 min). ESI drying gas (nitrogen) was set to 13 L/h, at 350 °C, with a nebulizer pressure of 35 psig; capillary voltage was set to 4.5 kV to favor fragmentation, and the optical parameters were set to 250, 225, and 60 V for the octopole radio frequency voltage (Oct RFV), fragmentor and skimmer, respectively. Runs were performed to acquire mass spectra in positive mode, and files were saved in profile mode, with an m/z range from 150 to 1500 m/z, and reading at 0.94 cycles per second, with a total of 10,000 transients per scan. Samples were injected in a random manner, and began with a set of 5 ‘dummy’ runs, where the same amount of a mix of all samples was injected.

#### Feature detection

Raw files were converted to CDF using Agilent’s Translator Utility (Agilent, CA, USA), and processing was done in the MZmine 2.15 platform. [63]. GridMass algorithm [64] was used for peak detection and base line correction was performed using an in-house implementation of a 2D–baseline correction method as a module for MZmine, which is available in http://bioinformatica.mty.itesm.mx/baseline2d. The baseline algorithm works by considering a range of time points from a window in m/z to reduce the background, which is estimated by a percentage of observed data within the window. This algorithm was run, considering an m/z window of 0.01, a retention time (RT) window of 1.5 min, and a 40% quantile; and peak detection via GridMass using a minimum height of 1000 counts, an m/z tolerance of 0.05, a RT window between 0.1 and 2.5 min, a smoothing time of 0.1 min and an intensity similarity ratio of 0.5. After feature detection, isotopic peaks grouping was performed using an m/z tolerance of 0.001 or 10 ppm, and a RT tolerance of 0.25, assuming a monotonic shape and a maximum charge of 2, with the lowest m/z as the most representative isotope. Alignment of features was achieved by the RANSAC algorithm with an m/z tolerance of 0.025 m/z, or 50 ppm, a RT tolerance of 2 min before and 1.5 min after RT correction, a minimum of 25% points matching the non-linear model below a threshold of 1 min. Finally, gap filling was performed using our own algorithm in R integrating the intensity over non-detected peaks in the RT window predicted using the detected peaks which better predicted the RT window of the detected features in the sample, with an m/z tolerance of 0.025 or 50 ppm. Further analysis of the resulting feature tables was performed in R, as stated in the Data Analysis section.

#### Grouping

Given the nature of the data, in which molecules may be confounded with isotopes of members of the same family, which is also rich on isomers, many of the detected features included isotopes and artifacts of the feature detection. Also, different adducts of the same molecule may be present, and, given the high voltage selected, molecules may be subject to fragmentation, which can yield information on their structure. Therefore, after processing the raw data with MZmine, an automated grouping procedure was performed in R. On the selected list of features, an in-house built algorithm was used to extract from the raw files information of the peaks in the samples, such as m/z, retention time, and intensity values from each measurement between the full width at quarter maximum (FWQM) of the chromatographic peak. Second, features were compared among each other, and if the retention times overlapped in some point, they were considered as “candidates” to belong to the same compound. Later, this candidate list was further trimmed based on the correlation of the respective intensities of the peaks at each time point, and were considered to belong to the same molecule only if their correlation was above 0.9. Since correlation rapidly degenerates as RT shifts, this threshold is equivalent to a shift of one scan (<1 s) even if the peak follows the exact same peak shape. An example of this grouping is shown and explained in Additional file 1: Figure S10. Selected peaks that belonged in the same group were considered as probably belonging to the same molecule, and were manually cleaned from isotopes by visually assessing the experimental mass spectra, and curated in search for adducts or fragments in the selected and automatically identified features.

#### Assignation of identity

Using the same information extraction algorithm from the grouping method (2.7.3) each peak was assigned a mean m/z measurement and its standard deviation, and was assigned a charge based on the first isotope. Monoisotopic masses of the most common adducts in positive mode for single ([M + H]^+^, [M + NH_4_] ^+^, [M + Na] ^+^, and [M + K] ^+^) and double charged (combinations of the previous adducts, e.g. [M + 2H] ^2+^, [M + H + NH_4_]^2+^, etc.) molecules were substracted from each feature mean m/z, and the resulting exact masses (4 for each single charged feature, 16 for double charged) were automatically searched using the Lipid MAPS® Representational State Transfer service [65] to access the Lipid MAPS® structure database [66]. The m/z window for the search was taken as 3 standard deviations of the m/z measurement, plus 25 ppm, and assignations were further cleaned by comparing the theoretical isotopic pattern of the molecular formula (including the adduct) with the experimental intensities, using as an allowed window the standard deviation of the intensity divided by the square of the correlation to the monoisotopic feature, plus 5% of the intensity value. Information retrieved included Molecular Formula, Name and Lipid MAPS® classification [45].

Therefore, a single feature could be assigned more than one putative molecular formula, each of which could in turn represent one or more known compounds. However, a set of different compounds that differ in identity, may belong to the same category in the Lipid MAPS® Lipid Classification System, having a similar biological role. Thus, using the main and secondary lipid classes, we estimated an “average” composition of each sample by estimating the proportion of ‘fixed’ and ‘approximate’ composition of all possible annotations for each mass. The ‘fixed’ composition represents the annotations that are independent of the choice of the assigned compound either because it contains only one compound or because all compounds are annotated as belonging to the same family. The ‘approximate’ composition represents a weighted average composition from all possible compound annotations. For instance, the single-charged feature 421.3–12.0 has an average m/z of 421.2893, and was assigned two possible molecular formulas: [C_25_H_40_O + H]^+^ and [C_27_H_42_O + K]^+^. These formulas have in total 12 possible assignations (1 and 11, respectively): one belonging at the second level to “Bile acids and derivatives [ST04]”, three to “Secosteroids [ST03]”, and eight to “Sterols [ST01]”. Therefore, it was annotated at the second level with an ‘approximate’ composition of 8.3% [ST04], 25% [ST03], and 66.7% [ST01]. However, since all those assignations fall within the category of “Sterol Lipids [ST]”, it was assigned as a ‘fixed’ [ST] at the first level. The analysis presented in the main manuscript correspond to the first level of annotations while the analysis presented in the supplementary material corresponds to curated assignations at the identity level.

#### Data analysis

All algorithms and statistical procedures, such as Analysis of Variance (ANOVA), t-tests, and Principal Component Analysis (PCA) were made using the R platform [67] with the *stats* library, unless otherwise stated. Grouping by Tukey’s Honestly Significant Difference (HSD) was done with the aid of the *agricolae* package [68]. For a result to be considered significant, a *p*-value threshold of 0.05 was set for the ANOVAs and t-tests; similarly, an α value of 0.05 was used for Tukey’s HSD. While linear model regressions were estimated using the R *stats* library, non-linear regression parameters were calculated using Microsoft Excel® (Microsoft Office Professional Plus 2013) “Add Trendline” function. Contents mentioned in the text are shown as mean and standard deviation unless otherwise stated. Flow cytometry data was accessed by use of the *flowCore* library [69], and purity assessed by predictive Linear Discriminant Analysis (pLDA) with the *DiscriMiner* library [70]*.* Direct access to MS files was achieved through *ncdf* package [71], and generation of theoretical exact mass and isotopic pattern calculations, using *Rdisop* library [72]. Previous to lipidomics analyses, the matrix with the raw intensities was quantile-normalized and log-transformed; then, centered and scaled feature-wise. All images here presented are of our own creation, using a combination of R, Microsoft Excel® and PowerPoint® (Microsoft Office Professional Plus 2013), and ACD/ChemSketch (ACD/Labs version 12.01, 2010) for chemical structures.

## Additional files

Additional file 1: Figures S1 to **S10**; **Table S1** to **S4**. This file contains a schematic explaining the terminology used in the article to refer to developmental stages (Additional file 1: **Figure S1**); information regarding acetogenin contents in mesocarp and seed of ‘Hass’ avocado fruit in a fresh weight basis (Additional file 1: **Figure. S2**, Tables 1 and 2); and micrographies of dyed idioblasts (Additional file 1: **Figure S3**), results from the assessment of extract purity (Additional file 1: **Figure S4**, **Table S3**), and acetogenin contents (Additional file 1: **Table S4**). Also, it contains complementary information on the metabolomics analysis, such as PCA of the mesocarp features (Additional file 1: **Figure S5**) and score plots of the mesocarp (Additional file 1: **Figure S6**) and idioblast (Additional file 1: **Figure S7**) samples. The corresponding part in seed tissue (Additional file 1: **Figure S8**) is included, along with the heatmaps of the manually curated TAGs, DAGs, and Acetogenins data (Additional file 1: **Figure S9**). Finally it contains an example of the grouping algorithm mentioned in the Methods section (Additional file 1: **Figure S10**). (DOCX 4325 kb)

Additional file 2: Tables S5 to **S7.** Manually curated features corresponding to abundant TAGs, DAGs and MAGs from ripening avocado mesocarp (Additional file 2: **Table S5**), idioblasts (Additional file 2: **Table S6**), and seeds (Additional file 2: **Table S7**); the latter also includes features with acetogenin signatures, along with their tentative assignations. (XLSX 66 kb)

## Abbreviations

AcO: Acetoxy
ACP: Acyl Carrier Protein
AMSL: Above Mean Sea Level
CoA: Coenzyme A
DAG: Diacylglycerol
DAPI: 4′,6-diamidino-2-phenylindole
DW: Dry Weight
ELSD: Evaporative Light Scattering Detector
ESI: Electrospray Ionization
FA: Fatty Acid
FW: Fresh Weight
HPLC: High Performance Liquid Chromatography
LC: Liquid Chromatography
LOX: linoleate:oxygen oxidoreductase
MAG: Monoacylglycerol
MS: Mass Spectrometry
NMR: Nuclear Magnetic Resonance
PCA: Principal Component Analysis
PUFA: Polyunsaturated Fatty Acid
RH: Relative Humidity
RT: Retention Time
TAC: Total Acetogenin Content
TAG: Triacylglycerol
TOF: Time Of Flight
Tukey HSD: Tukey’s Honestly Significant Difference
UPA: Unknown Putative Acetogenin
VWD: Variable Wavelength Detector
