# Supplementary material for: Avocado fruit maturation and ripening: dynamics of aliphatic acetogenins and lipidomic profiles from mesocarp, idioblasts and seed
Source: BMC Plant Biol. 2017 Sep 29;17:159. doi: 10.1186/s12870-017-1103-6 (PMC5623960; doi:10.1186/s12870-017-1103-6)
Supplement: Supplementary file 1 — to S10; Table S1 to S4. This file contains a schematic explaining the terminology used in the article to refer to developmental stages (Additional file 1: Figure S1); information regarding acetogenin contents in mesocarp and seed of ‘Hass’ avocado fruit in a fresh weight basis (Additional file 1: Figure. S2, Tables 1 and 2); and micrographies of dyed idioblasts (Additional file 1: Figure S3), results from the assessment of extract purity (Additional file 1: Figure S4, Table S3), and acetogenin contents (Additional file 1: Table S4). Also, it contains complementary information on the metabolomics analysis, such as PCA of the mesocarp features (Additional file 1: Figure S5) and score plots of the mesocarp (Additional file 1: Figure S6) and idioblast (Additional file 1: Figure S7) samples. The corresponding part in seed tissue (Additional file 1: Figure S8) is included, along with the heatmaps of the manually curated TAGs, DAGs, and Acetogenins data (Additional file 1: Figure S9). Finally it contains an example of the grouping algorithm mentioned in the Methods section (Additional file 1: Figure S10). (DOCX 4325 kb) [file 12870_2017_1103_MOESM1_ESM.docx]

**Additional file 1**


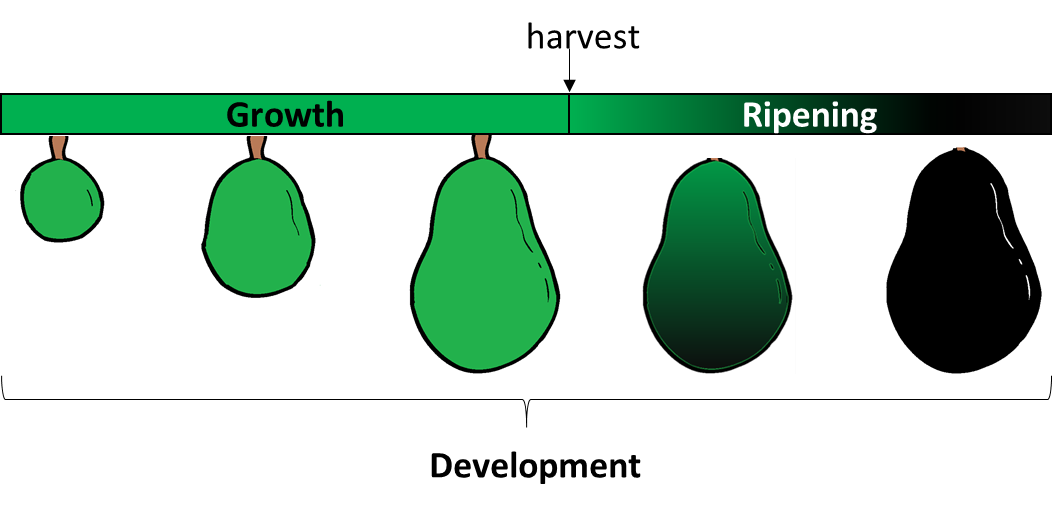


**Figure S1. Avocado growth and ripening.** A schematic is shown, as to explain the terminology used in the article. Avocado growth is referred to the change in size as fruit expands while the still attached to the tree, and, after harvesting, ripening is used to make reference to the processes occurring from detachment until the fruit is ready to eat. Both growth and dry weight of the fruit are proxies for maturation, while softening and color are for the ripening stages. The entire process is described as fruit development, encompassing the entire life cycle of the fruit.


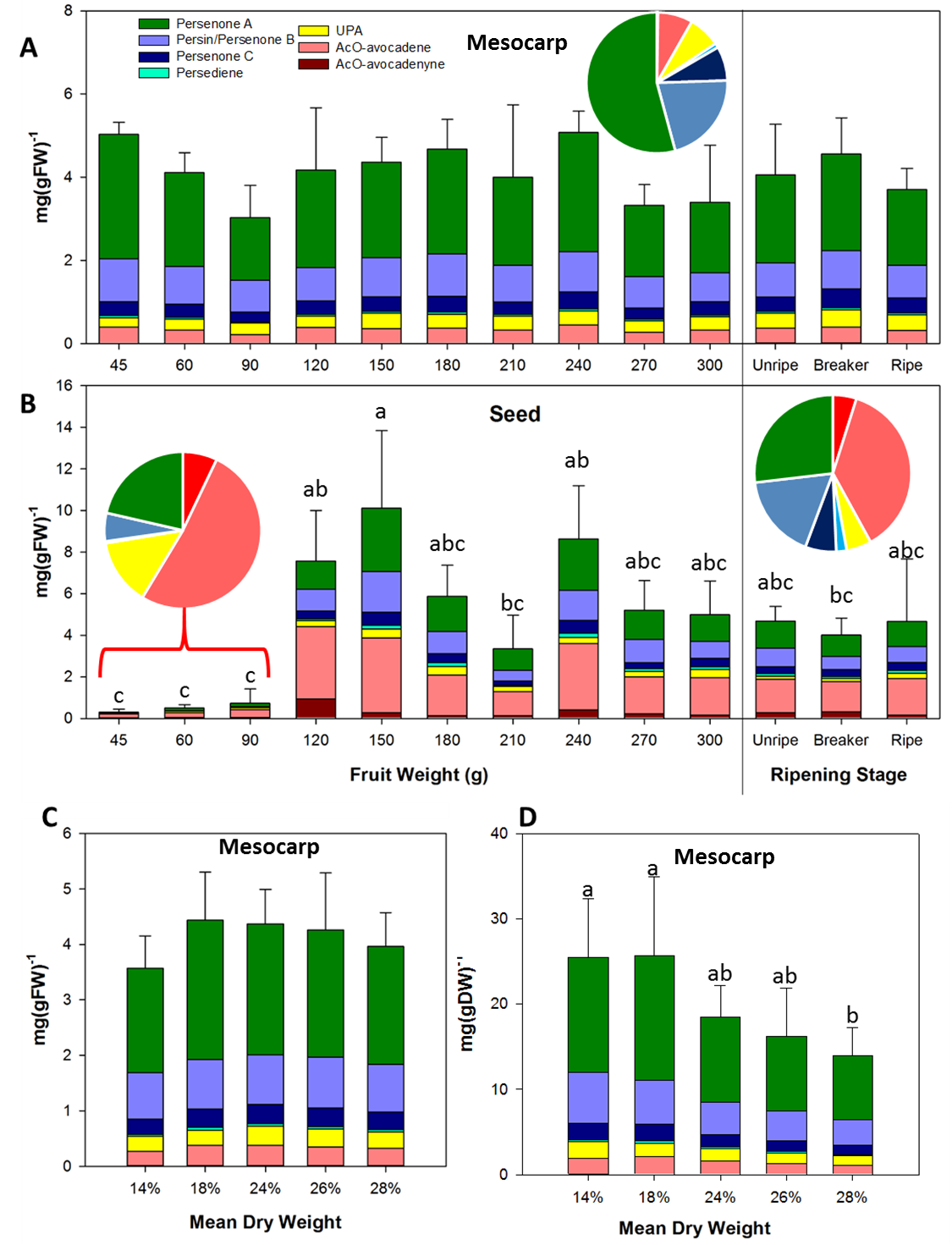


**Figure S2. Acetogenin contents in mesocarp and seed of ‘Hass’ avocado fruit during development.** (**A**) Acetogenin concentrations, in a fresh weight basis, during maturation (left) and postharvest ripening stages (right) of avocado mesocarp and (**B**) seed tissue, grouped by fruit fresh weigth (n=3), and Acetogenin concentrations in avocado mesocarp, excluding ripening, grouped by dry weight means (n=6), showing concentration as fresh (**C**) and dry weight (**D**). Means show the average of the corresponding biological replicates, and error bars reflect the standard deviation of the Total Acetogenin Concentrations (TAC); letters denote homogeneous group in a Tukey HSD test (α<0.05.)

**
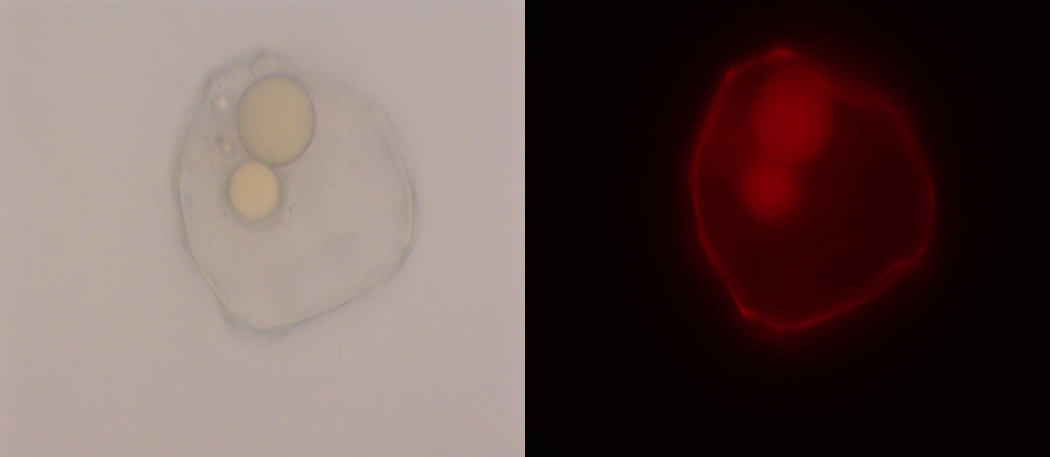
**

**Figure S3. Developing idioblast.** Micrography of an idioblast with a partially filled sac, dyed with Nile Red, taken under bright field (left), and a red (right) filter using fluorescence microscopy.


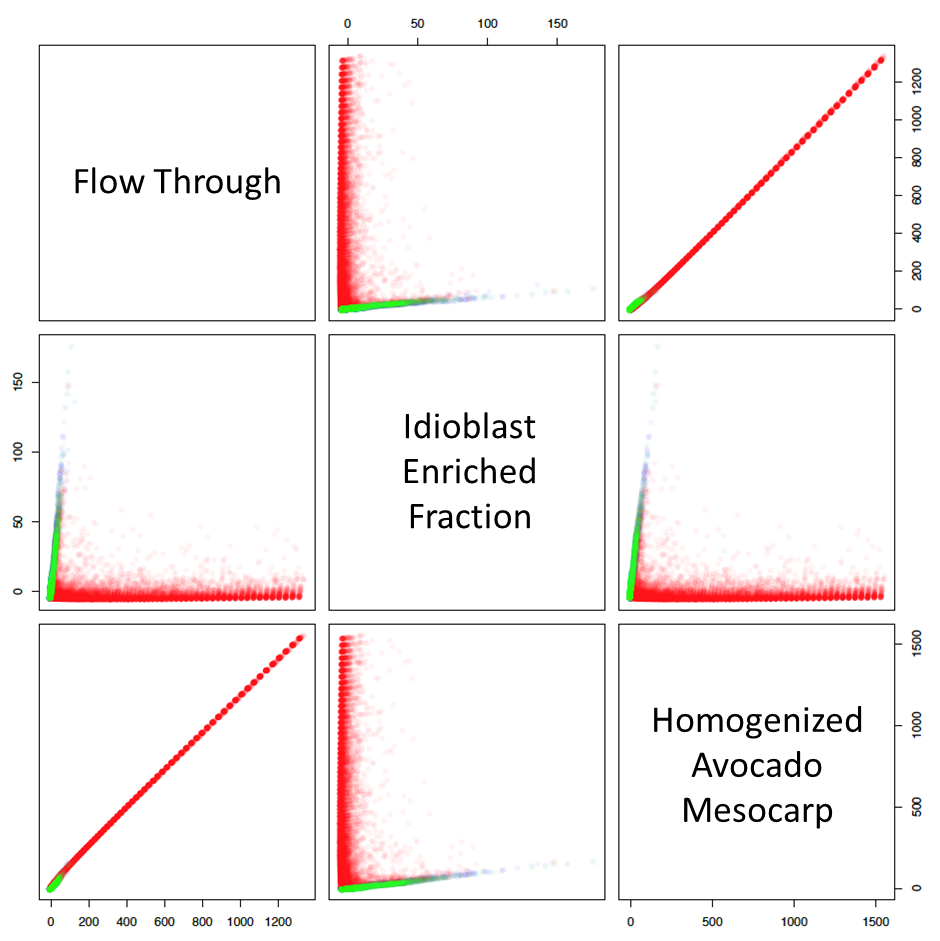


**Figure S4.** Red-Nile stained flow through (blue dots), idioblasts preparations (red dots), and homogenized avocado mesocarp (green dots) distribution assessed by flow cytometer, as classified using a Linear Discriminant Analysis.

**
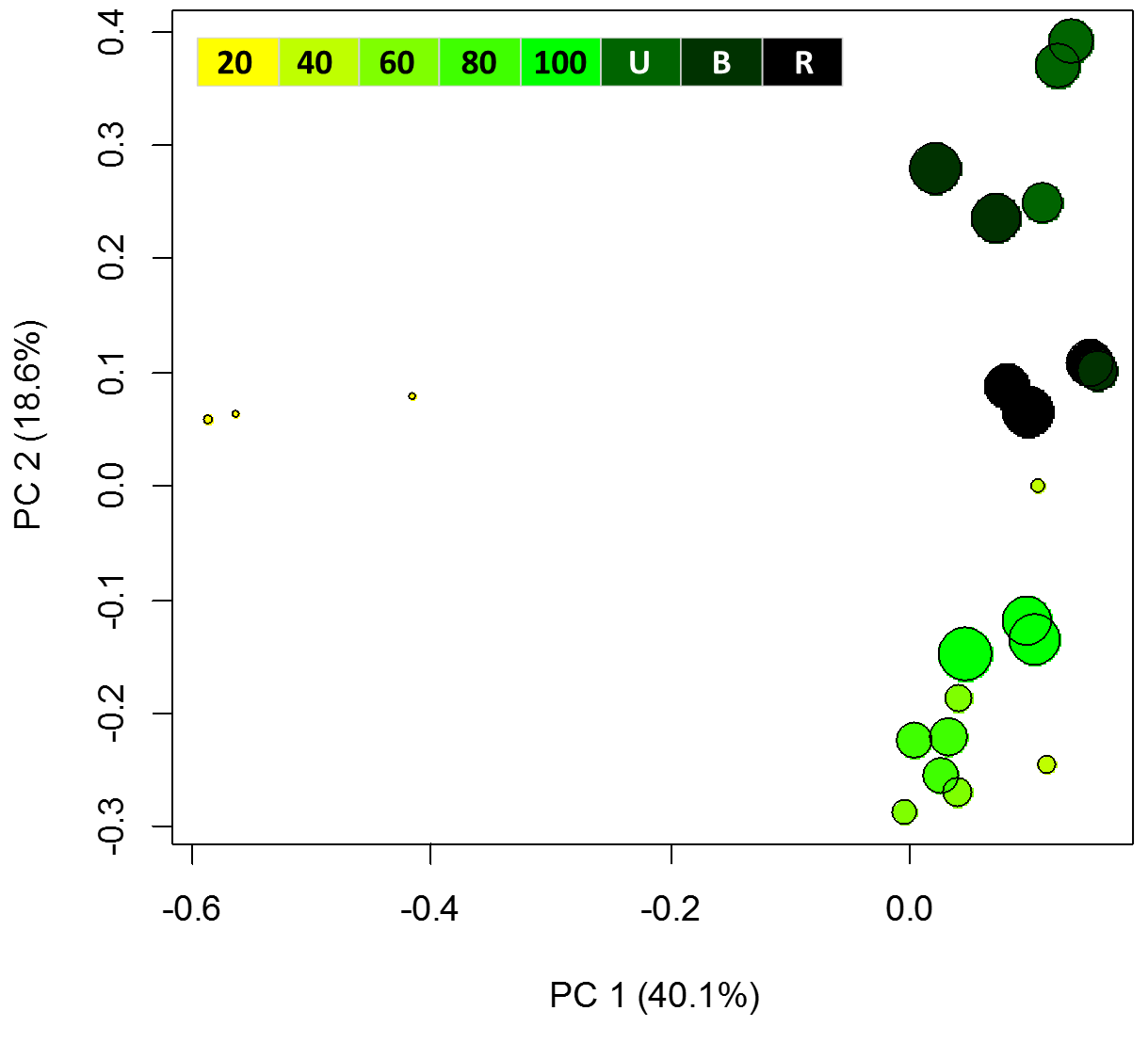
**

**Figure S5.** PCA analysis of features detected in all mesocarp samples. Fruit growing and ripening stage is indicated by color and mesocarp DW is depicted by circle size.


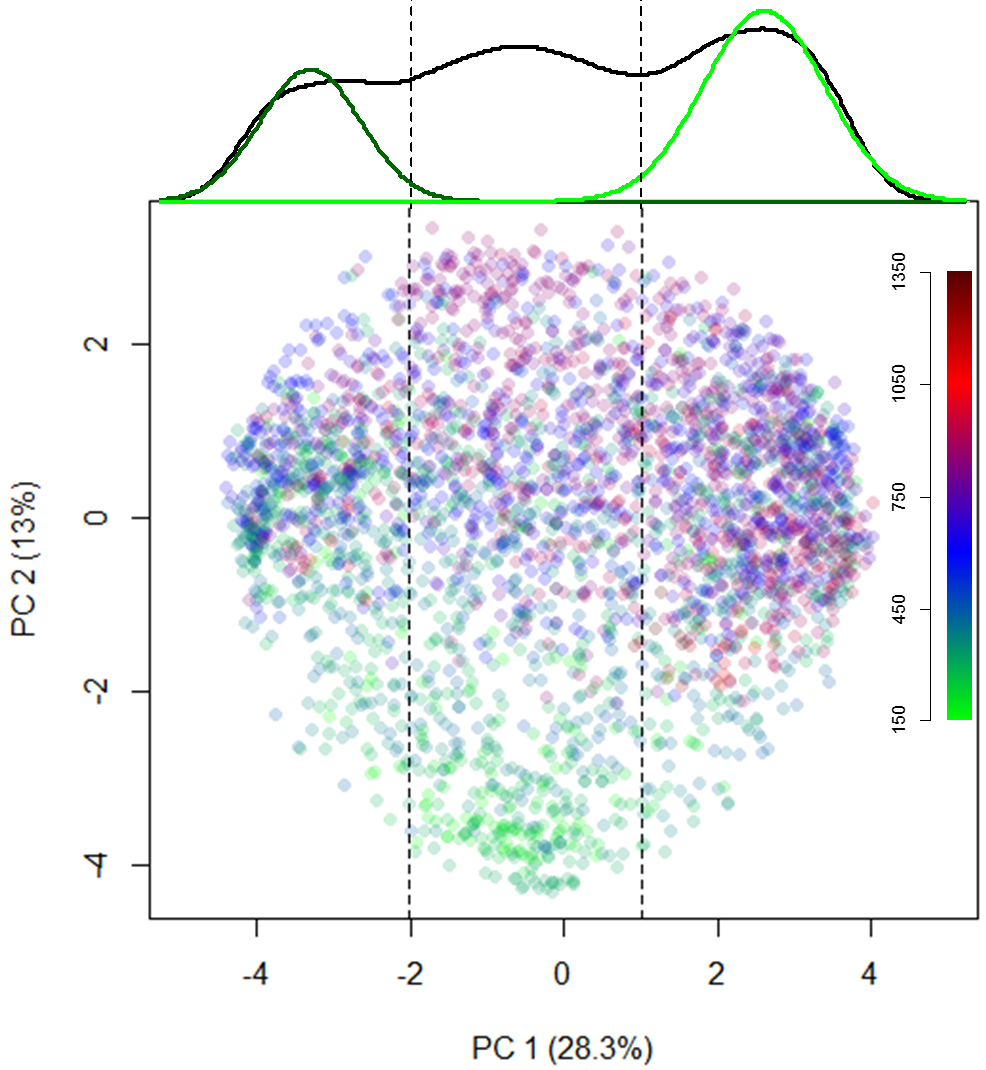


**Figure S6.** Score plot showing features detected in all mesocarp samples, except the smallest stage (60g). Color is set by m/z value in a continuous scale from green, to blue, to red, as mass increases. Kernel density estimates on the first component are shown above the plot, with the corresponding Gaussian regressions used to determine the most important features to be used (right and left-most populations).

**
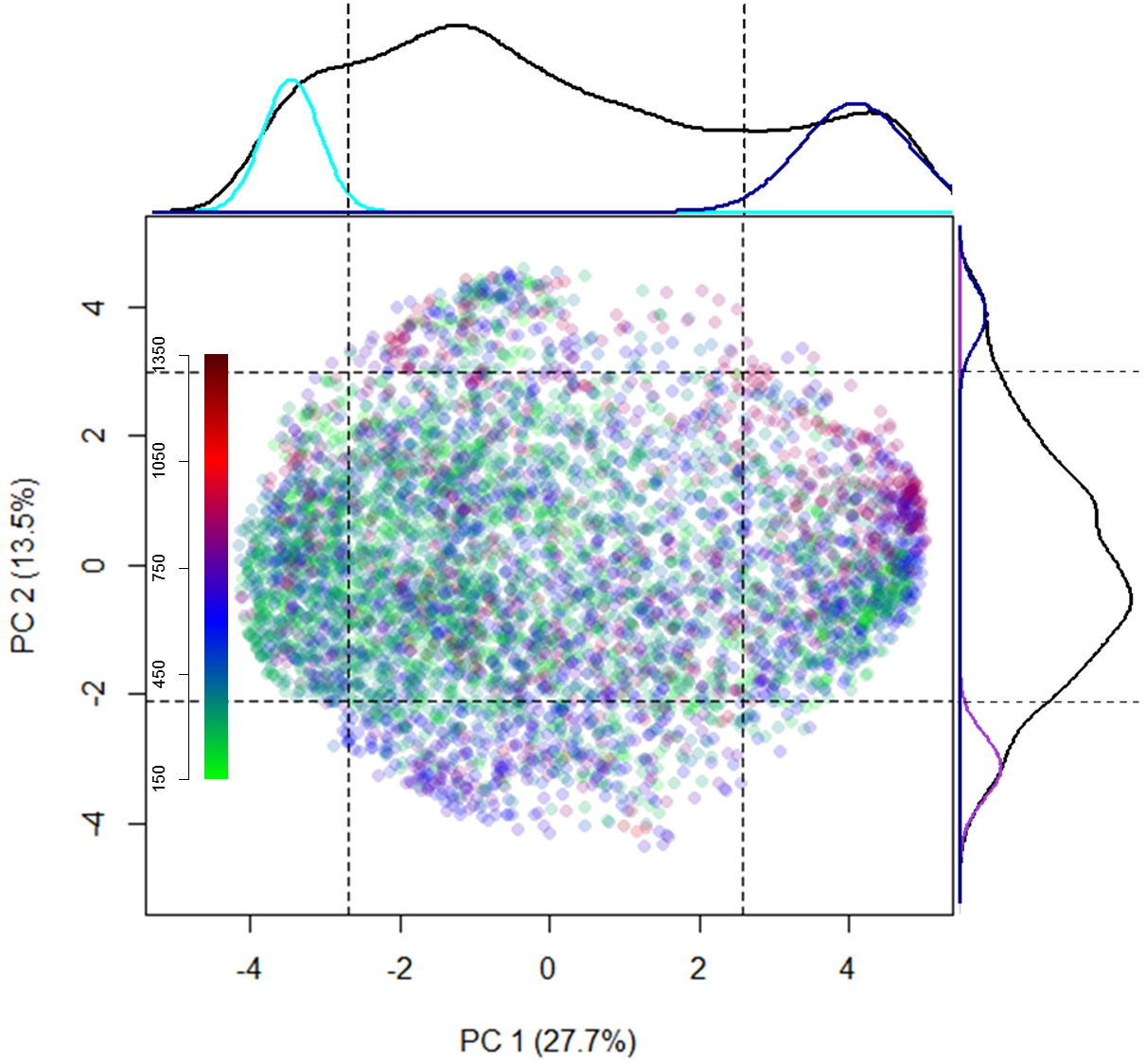
**

**Figure S7**. Score plot showing features detected in all idioblasts samples. Color is set by m/z value in a continuous scale from green, to blue, to red, as mass increases. Kernel density estimates on the two components are shown above the plot, with the corresponding Gaussian regressions used to determine the most important features to be used (upper and lower-most populations).


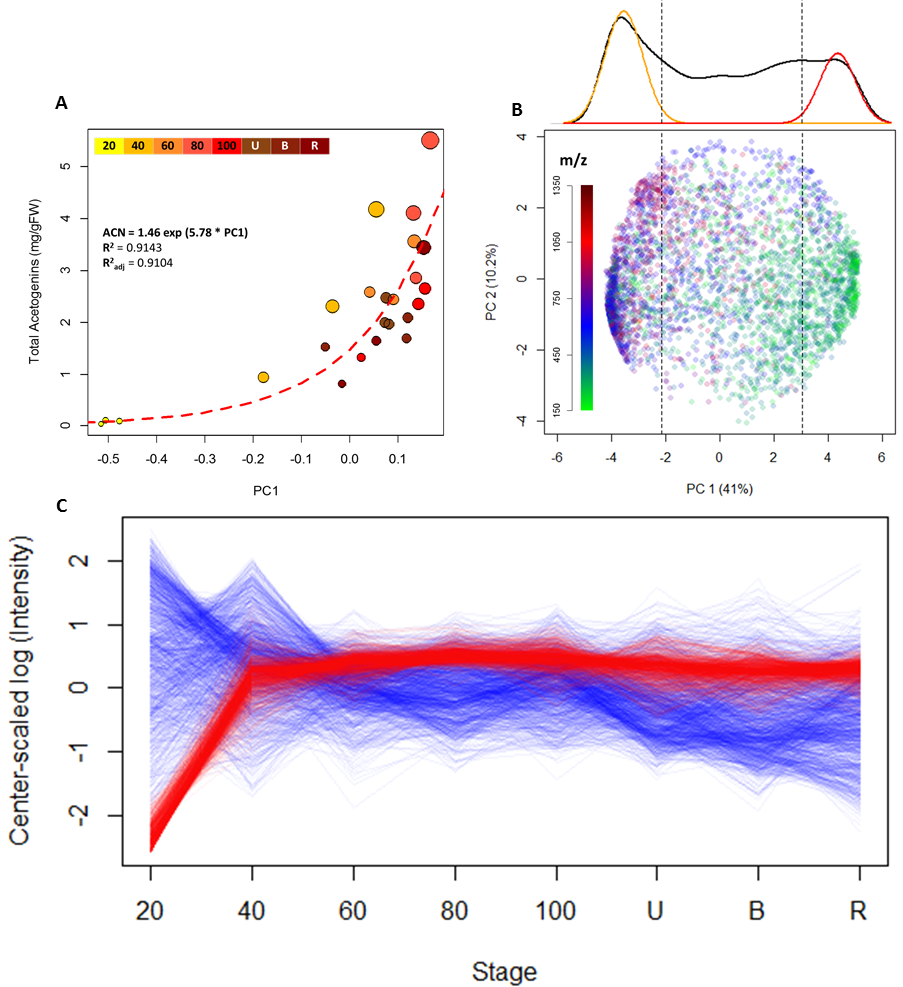


**Figure S8**. **(A)** Total acetogenin content by dry weight as a function of the principal component. Color indicates maturation or ripening stage and size of the dot represents acetogenin content by dry weight. A linear regression showing the high correlation is shown. **(B)** Score plot showing features detected in all idioblasts samples. Color is set by m/z value in a continuous scale from green, to blue, to red, as mass increases. Kernel density estimates on the main component are shown above the plot, with the corresponding Gaussian regressions used to determine the most important features to be used (right and left-most populations). (**C**) Mean values (n=3) as a function of fruit growth and ripening stage of the two divergent groups of selected features from PC1 **(B)**, red lines represent TAC-related features, which are abundant in mature seeds, and blue lines represent high molecular weight compounds which are abundant in immature seeds.

**TAGs DAGs**

**
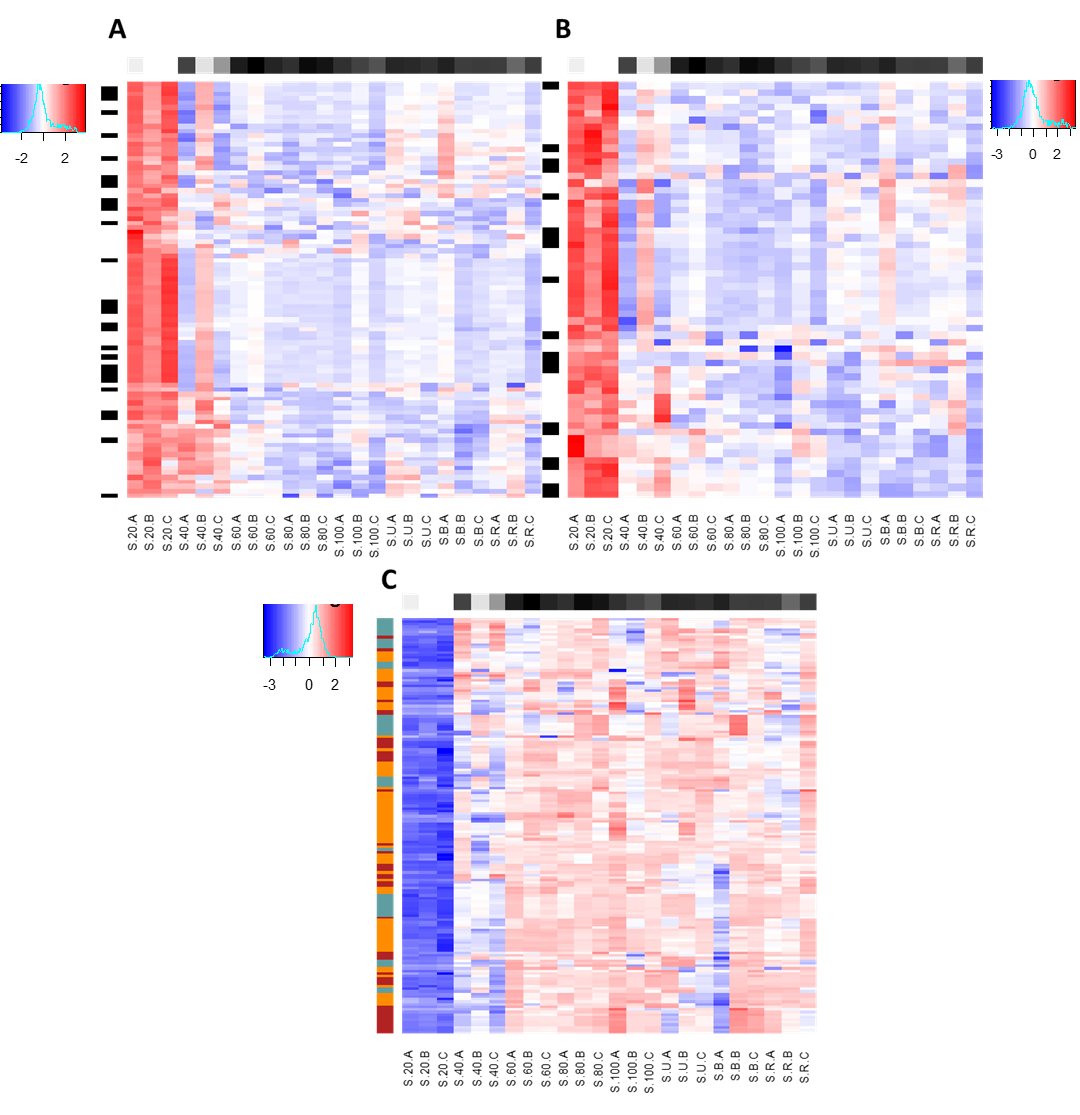
**

**Acetogenins**

**Figure S9**. Seed TAGs, DAGs and acetogenin dynamics through fruit development. Heatmaps of log-transformed, center-scaled intensities of features with manually curated identities corresponding to **(A)** TAGs and **(B)** DAGs, in which the strip to the lefts marks odd-chain moieties (in black), and **(C)** Acetogenins, in which the strip to the left marks the carbon number: 17, turquoise; 19, red; and 21, orange. In all cases, concentration goes from lowest (blue) to highest (red) and the horizontal strip at the top denotes dry weight in a gradient from white to black.


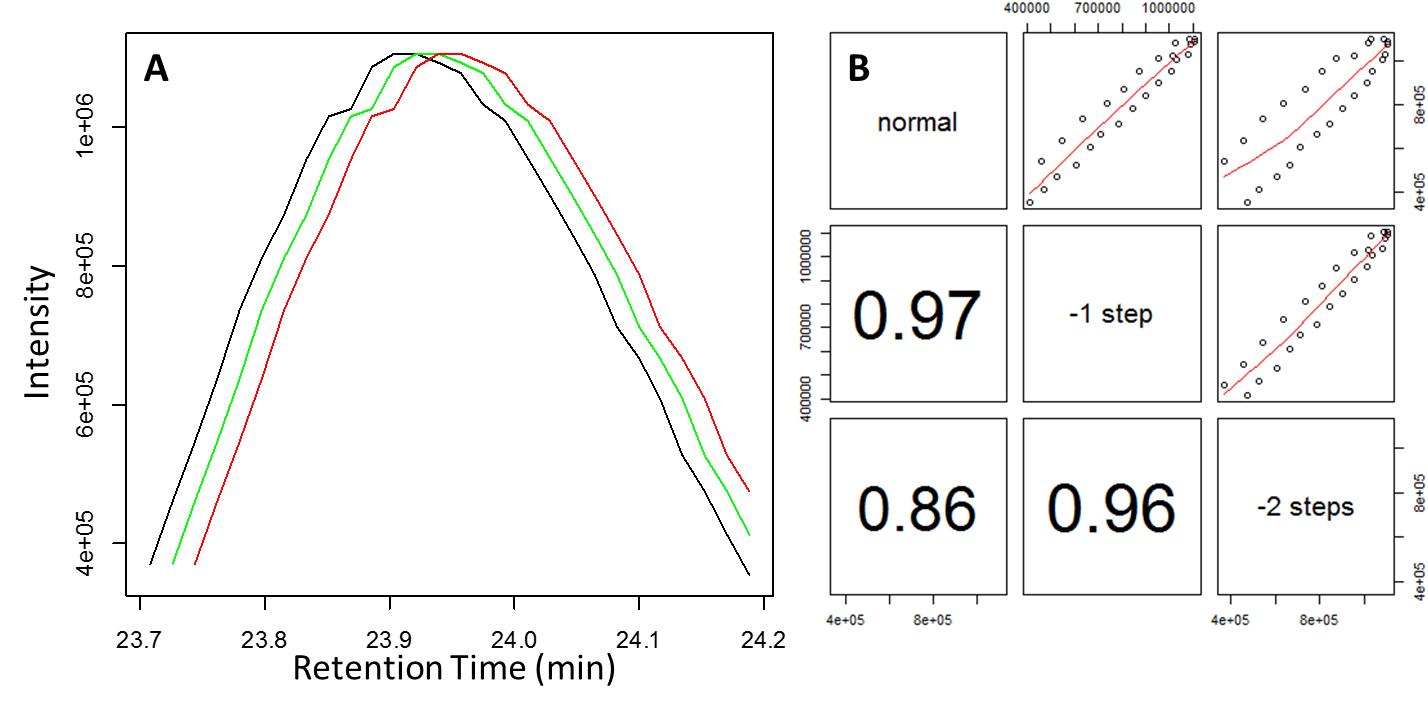


Figure S10. Example of the grouping algorithm performance on a RT shifted peak. (A) A randomly chosen chromatographic peak, as originally measured (black) and shifted one (green) and two (red) seconds in RT. (B) Pairs plot showing raw data (upper) and Pearson correlations (lower diagonal) between each peak showing that correlation rapidly degenerates with RT shifts; the set threshold translates to a difference of 2 scans (roughly 2.1 seconds) in the best-case scenario, where the peak is the exactly the same: the peak in green has a correlation of 0.97 and it is classified as the same group the black peak, while the peak in red would be not, due to a correlation of 0.86. Therefore, this function is able to differentiate closely eluting compounds, which would be impossible using existing feature list-based algorithms, and it is presumed to correctly assign fragments, adducts and isotopes. Finally, samples were cleaned from isotopes and duplicate peaks in a group-wise fashion, to avoid deleting closely eluting, related compounds.

**Table S1. Acetogenin content of mesocarp.** Individual acetogenin content is shown as mean ± standard deviation (n=3) for the measured acetogenins, as milligrams of each acetogenin per gram fresh weight.

| Stage | AcO-avocadyne | AcO-avocadene | UPA | Persediene | Persenone C | Persenone A | Persenone B | TOTAL |
| --- | --- | --- | --- | --- | --- | --- | --- | --- |
| 45 g | 0.01±0 | 0.39±0.02 | 0.23±0.05 | 0.05±0 | 0.33±0.03 | 2.99±0.39 | 1.03±0.03 | 5.03±0.29 |
| 60 g | 0.01±0 | 0.31±0.04 | 0.27±0.07 | 0.04±0.01 | 0.31±0.04 | 2.25±0.48 | 0.92±0.05 | 4.11±0.48 |
| 90 g | 0.01±0 | 0.2±0.03 | 0.28±0.01 | 0.03±0 | 0.24±0.05 | 1.5±0.49 | 0.76±0.22 | 3.03±0.78 |
| 120 g | 0.01±0 | 0.37±0.18 | 0.27±0.07 | 0.05±0.02 | 0.33±0.12 | 2.34±1 | 0.8±0.18 | 4.17±1.5 |
| 150 g | 0.01±0 | 0.35±0.09 | 0.37±0.03 | 0.04±0.01 | 0.35±0.04 | 2.3±0.4 | 0.94±0.09 | 4.36±0.6 |
| 180 g | 0.01±0 | 0.35±0.05 | 0.34±0.05 | 0.05±0 | 0.38±0.05 | 2.52±0.46 | 1.02±0.16 | 4.67±0.71 |
| 210 g | 0.01±0 | 0.31±0.13 | 0.34±0.11 | 0.04±0.02 | 0.3±0.13 | 2.12±1.01 | 0.88±0.35 | 4±1.74 |
| 240 g | 0.01±0 | 0.44±0.1 | 0.33±0.02 | 0.05±0.01 | 0.4±0.06 | 2.87±0.39 | 0.97±0.07 | 5.08±0.51 |
| 270 g | 0.01±0 | 0.26±0.03 | 0.28±0.06 | 0.03±0.01 | 0.27±0.05 | 1.71±0.25 | 0.76±0.11 | 3.33±0.49 |
| 300 g | 0.01±0 | 0.31±0.15 | 0.33±0.13 | 0.04±0.02 | 0.32±0.16 | 1.7±0.7 | 0.69±0.23 | 3.4±1.37 |
| Unripe | 0.01±0.01 | 0.35±0.1 | 0.36±0.04 | 0.04±0.01 | 0.35±0.07 | 2.12±0.85 | 0.82±0.26 | 4.06±1.22 |
| Breaker | 0.02±0 | 0.37±0.06 | 0.42±0.04 | 0.05±0.01 | 0.44±0.09 | 2.33±0.51 | 0.92±0.21 | 4.56±0.87 |
| Ripe | 0.01±0 | 0.3±0.08 | 0.38±0.03 | 0.04±0.01 | 0.36±0.05 | 1.82±0.28 | 0.79±0.11 | 3.71±0.5 |

**Table S2. Acetogenin contents of seed.** Individual acetogenin content is shown as mean ± standard deviation (n=3) for the measured acetogenins, as milligrams of each acetogenin per gram fresh weight.

| Stage | AcO-avocadyne | AcO-avocadene | UPA | Persediene | Persenone C | Persenone A | Persenone B | TOTAL |
| --- | --- | --- | --- | --- | --- | --- | --- | --- |
| 45 g | 0.03±0.01 | 0.17±0.08 | 0.05±0.03 | 0±0 | 0±0 | 0.04±0.02 | 0±0 | 0.3±0.15 |
| 60 g | 0.04±0.02 | 0.24±0.07 | 0.06±0.03 | 0±0 | 0±0 | 0.12±0.05 | 0.03±0.01 | 0.49±0.17 |
| 90 g | 0.04±0.03 | 0.38±0.31 | 0.09±0.07 | 0.01±0.01 | 0±0 | 0.17±0.19 | 0.06±0.08 | 0.74±0.68 |
| 120 g | 0.92±0.55 | 3.49±1.14 | 0.29±0.11 | 0.09±0.07 | 0.37±0.07 | 1.35±0.54 | 1.05±0.41 | 7.56±2.43 |
| 150 g | 0.28±0.22 | 3.59±1.34 | 0.44±0.21 | 0.18±0.03 | 0.61±0.11 | 3.04±1.23 | 1.96±1.15 | 10.11±3.74 |
| 180 g | 0.13±0.06 | 1.95±0.46 | 0.42±0.07 | 0.19±0.06 | 0.42±0.12 | 1.69±0.46 | 1.08±0.32 | 5.87±1.49 |
| 210 g | 0.13±0.06 | 1.16±0.62 | 0.25±0.11 | 0.06±0.02 | 0.2±0.11 | 1.03±0.55 | 0.53±0.24 | 3.34±1.62 |
| 240 g | 0.39±0.06 | 3.2±1.17 | 0.29±0.15 | 0.22±0.04 | 0.6±0.11 | 2.48±0.8 | 1.44±0.53 | 8.63±2.55 |
| 270 g | 0.22±0.07 | 1.77±0.57 | 0.27±0.04 | 0.12±0.08 | 0.29±0.1 | 1.41±0.39 | 1.11±0.52 | 5.19±1.43 |
| 300 g | 0.15±0.11 | 1.8±0.56 | 0.41±0.23 | 0.12±0.03 | 0.42±0.15 | 1.28±0.51 | 0.81±0.42 | 4.99±1.61 |
| Unripe | 0.28±0.02 | 1.6±0.39 | 0.15±0.05 | 0.14±0.03 | 0.32±0.05 | 1.31±0.35 | 0.9±0.23 | 4.69±0.7 |
| Breaker | 0.31±0.14 | 1.47±0.42 | 0.15±0.07 | 0.09±0.03 | 0.34±0.15 | 1.02±0.26 | 0.64±0.02 | 4±0.81 |
| Ripe | 0.16±0.11 | 1.78±1.25 | 0.25±0.09 | 0.15±0.14 | 0.37±0.2 | 1.2±0.94 | 0.76±0.39 | 4.65±3.02 |

**Table S3.** Results from a LDA classification of cell cytometry runs of Nile Red stained flow through, idioblast enriched fraction and homogenized mesocarp.

|  | Predicted fraction | | |  |
| --- | --- | --- | --- | --- |
| Original fraction | Flow through | Idioblast Enriched Fraction | Homogenized Mesocarp | Total cell count |
| Flow through | 8021 | 67 | 1912 | 10000 |
| Idioblast enriched fraction | 962 | 8792 | 246 | 10000 |
| Homogenized mesocarp | 1715 | 64 | 8221 | 10000 |

**Table S4. Acetogenin contents of Idioblasts.** Individual acetogenin content is shown as mean ± standard deviation (n=3) for the measured acetogenins, as milligrams of each acetogenin per gram fresh weight.

| Stage | AcO-avocadyne | AcO-avocadene | UPA | Persediene | Persenone C | Persenone A | Persenone B | TOTAL |
| --- | --- | --- | --- | --- | --- | --- | --- | --- |
| 45 g | 0.01±0 | 0.15±0 | 0.09±0.02 | 0.02±0 | 0.13±0.02 | 1.09±0.1 | 0.39±0.02 | 1.88±0.05 |
| 120 g | 0.01±0 | 0.14±0.08 | 0.1±0 | 0.02±0.01 | 0.12±0.05 | 0.85±0.57 | 0.27±0.1 | 1.52±0.8 |
| 180 g | 0.01±0 | 0.1±0.01 | 0.1±0.02 | 0.01±0 | 0.1±0.02 | 0.59±0.17 | 0.24±0.05 | 1.15±0.27 |
| 240 g | 0.01±0 | 0.15±0.03 | 0.1±0.01 | 0.02±0 | 0.12±0.02 | 0.8±0.1 | 0.28±0.04 | 1.48±0.17 |
| 300 g | 0.01±0 | 0.13±0.05 | 0.13±0.04 | 0.01±0.01 | 0.12±0.03 | 0.56±0.18 | 0.22±0.07 | 1.18±0.36 |
| Unripe | 0.01±0 | 0.21±0.08 | 0.2±0.04 | 0.02±0.01 | 0.19±0.03 | 1±0.29 | 0.42±0.15 | 2.05±0.56 |
| Breaker | 0.01±0 | 0.24±0.11 | 0.3±0.09 | 0.04±0.02 | 0.31±0.13 | 1.48±0.78 | 0.63±0.26 | 2.99±1.37 |
| Ripe | 0.01±0 | 0.29±0.08 | 0.39±0.02 | 0.04±0.01 | 0.37±0.05 | 1.78±0.3 | 0.77±0.08 | 3.64±0.52 |
